# Supplementary material for: Effects of public-health measures for zeroing out different SARS-CoV-2 variants
Source: Nat Commun. 2023 Aug 29;14:5270. doi: 10.1038/s41467-023-40940-4 (PMC10465600; doi:10.1038/s41467-023-40940-4)
Supplement: Supplementary file 1 — Supplementary Information [file 41467_2023_40940_MOESM1_ESM.pdf]

Supplementary information

A. Tracking COVID-19 outbreaks and interventions

A.1 Data collection for outbreaks and public-health measures

From 1 April 2020 to 31 May 2022, we collected daily reports of COVID-19 cases and suspected sources, start and end dates, and strains of 261 outbreaks in the mainland of China (23 provinces, 5 autonomous regions, and 4 municipalities) (see supplementary Figure 1). The data was obtained from local government websites, reports from the local Centers for Disease Control and Prevention, as well as updates from official social media accounts of local authorities or health departments. The start date was marked by the first reported index case, while the end date was determined by the last reported case for each outbreak. Additionally, we recorded the number of daily new infections identified among close contacts who had been isolated and quarantined during the outbreaks, based on available records reported by local governments. In order to have an adequate sample size for evaluating the effects of interventions, we excluded outbreaks that involved fewer than 50 cases or lasted for less than 7 days. As a result, there are 131 outbreaks that were eventually included in this study.

Further, the daily data of nine public-health measures for 131 outbreaks were also collected in this study, including stay-at-home order (SO), business premises closures (BPC), public transportation closures (PTC), gathering restrictions (GR), workplace closures (WC), school closures (SC), facial masking (FM), mass screening (MS) using Polymerase chain reaction (PCR) tests, medicine management (MM), and contact tracing

(CT) (supplementary Table 1). We coded the stringency for each measure under the zero-COVID policy in China from no interventions (0) to the strictest level represented by the highest ordinal value, such as the lockdown of the whole city (stay-at-home mandate). As the implementation of COVID-19 intervention policy might vary in stringency across geographic areas/administrative division within a city (prefecture level), we also used another ordinal metric to denote the geographic range (1 - Administrative divisions level, 2 - Township level, 3 - District/county level, 4 - City/prefectures level) for BPC, PTC, GR, WC, SC and MM measures, where the most stringent NPI was presented. During the pandemic, cities in China typically manage administrative divisions or villages as the basic units. Several geographically adjacent administrations/villages form a street or township, and several neighboring streets/townships constitute a district or county. Cities/prefectures usually encompass multiple urban districts and surrounding counties. Therefore, a higher value of the geographic scope indicator means that a larger area or population would be affected by the measures implemented.

Furthermore, we documented the temporal frequency of MS as an integer ranging from 1 to 7, denoting the number of tests for each person per week. CT was recorded on a continuous scale, described as the ratio of the number of cases detected in isolated close contacts to the total number of cases in daily reports, representing the intensity of contact tracing. To avoid overestimating the close contacts actually tracked, CT was multiplied by a confounding factor of 0.8, indicating an upper limit on tracking capacity. Please note that we only focused on how to achieve the initial containment by implementing different measures when a new outbreak occurs. Therefore, this study did not include the measures that had been implemented consistently for preventing reintroduction of the virus during

the study period in China, such as inter-city and international travel restrictions and quarantine for incoming travellers from other countries or other high-risk Chinese cities with ongoing community transmission [1,2]. To ensure accuracy, the intervention data for each outbreak were collected independently by two authors, and then the data were cross checked and decided following discussion between the authors, with full agreement required prior to inclusion. Datasets and detailed sources are available via https://github.com/wxl1379457192/Zeroing_out_emerging_contagions/blob/main/Dataset.zip.

Considering NPIs as binary indicator variables have been commonly used, which can help us to quantify the stringency and effects of measures [3–5]. However, outbreaks were reported at the city or prefectural level, but interventions were often deployed at a smaller administrative unit such as community or district level in China. Moreover, when quantification was based on the ordinal levels of NPIs, we encountered situations where specific geographic areas or intensity policies only existed during certain periods of variant circulations. This makes it challenging to compare the effects of one policy across different time periods. For example, Medicine Management measures of mid-stringency (Require PCR tests for purchasers) were implemented after the emergence of the Delta variant. Therefore, to longitudinally evaluate the impact of NPIs on variants, we assembled the data of deployment geographical scope, thus combining them with the stringency of NPIs and transforming the space-varying interventions into a variable ranging from 0 to 1.

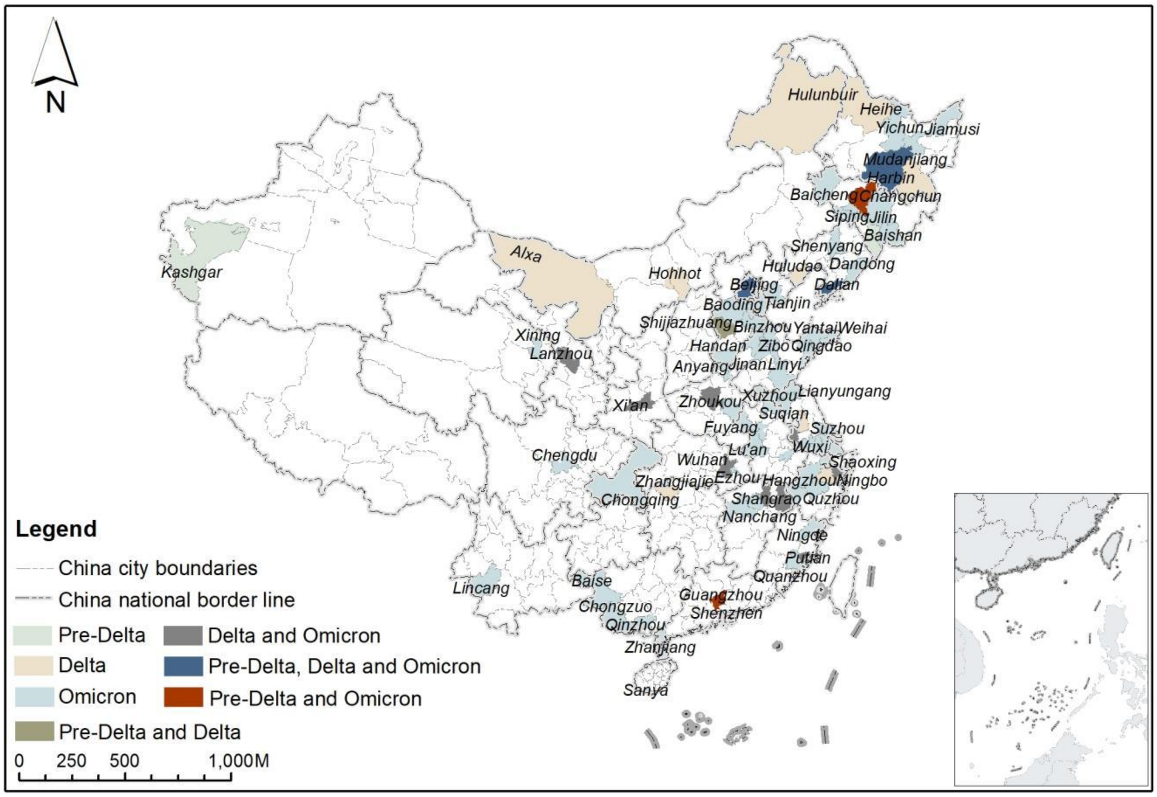

Supplementary Fig 1. Spatial distribution of the 131 study outbreaks, with colours representing the predominant variants. Areas with multiple outbreaks of multi-variants were also marked by red (pre-Delta and Delta variants), green (pre-Delta and Omicron variants), pink (Delta and Omicron variants), and blue (pre-Delta, Delta and Omicron variants) separately.

Supplementary Table 1. Data of public-health measures for each outbreak collected in this study.

ID	Name	Description	Measurement	Coding of stringency

SO	Stay-at-home order	Record orders to restrict the movement of people within the certain regions, with residents required to stay at home or government-approved self-isolation sites except for essential needs like medical appointments.	Ordinal scale	0-No measures<br><br>1-Require not leaving house or assisted self-isolation site for contacts with high risk of exposure<br><br>2-Require not leaving house for compounds with high risk of exposure<br><br>3-Require not leaving house for subdistricts with high risk of exposure<br><br>4-Require not leaving house for districts or county with high risk of exposure<br><br>5-Require not leaving house for the whole city (only urban guaranteed personnel can leave)
BPC	Business premises closure	Record orders to the temporary access control or shutdown of non-essential businesses or public places, like shopping malls, cinemas, bars, restaurants and other entertainment venues. The access control or restriction is usually implemented with the help of health codes, which designate individuals as green, yellow, or red based on their potential exposure to the virus.	Ordinal scale	0-No measures<br><br>1-Require health code<br><br>2-Require restricted numbers of visitors<br><br>3-Require closing some entertainment place (e.g., cinemas, karaoke houses, underground natatoria, etc)<br><br>4-Require closing all business premises (except grocery store, medical institution, etc)

PTC	Public transportation closure	Record orders to the routine environmental disinfection, boarding control and temporary suspension or reduction of public transportation services. In some cases, entire regions or cities were placed under strict lockdown, and all public transportation services were suspended. This meant that buses, subways and other forms of public transportation were not running. In other areas, less severe measures were implemented, such as limiting the frequency or capacity of public transportation services, or requiring commuters to provide proof of a negative COVID-19 test or green health code before boarding.	Ordinal scale	0-No measures<br><br>1-Require environmental disinfection<br><br>2-Require health code or restricted number of passengers<br><br>3-Reduce volume/route of transport available<br><br>4-Require closing public transport
GR	Gathering restriction	Record orders on limiting the number of people who could gather in public or private spaces to slow the spread of the virus. In high-risk areas, gathering restrictions were often implemented as limiting gatherings to fewer than 10 people, or banning all public gatherings entirely. In other areas, less severe measures were implemented, such as limiting the capacity of public venues like restaurants, cinemas, and shopping malls, or recommending people to maintain a certain distance from each other in public spaces. In addition to these public gathering restrictions, there were also restrictions on private gatherings, particularly during holidays and	Ordinal scale	0-No restrictions<br><br>1-Recommend restriction<br><br>2-Restriction on gathering between 11-500 people<br><br>3-Restriction on gathering of 10 people or less

		family gatherings when people are more likely to gather in large groups.		
WC	Workplace closure	Record the temporary shutdown of non-essential business, and the limitation on the number of employees allowed to work on-site. This means that some employees may be required or recommended to work from home depending on the specific measures implemented, followed by some social distancing measures in the workplace, or facial mask wearing requirement and other personal protective equipment.	Ordinal scale	0-No measures<br>1-Recommend work from home<br>2-Require work from home for all-but-essential workplaces
SC	School closure	Record closings of some certain or all levels of schools, from kindergarten to universities. During the school closures, students were required to stay home and continue their studies through online or remote learning platforms. The government also worked with schools to implement safety measures, such as regular disinfection of classrooms and requiring students to wear masks while on campus	Ordinal scale	0-No measures<br>1-Require closing schools at certain levels (e.g., elementary school, middle school, high school, etc)<br>2-Require closing all levels
FM	Facial masking	Record policies for encouraging or requiring mask use outside the home, especially in high-risk areas such as hospitals, public transportation, and crowded places. As the outbreak continued, the policy would also be expanded to require the use of masks in all public places, including shopping malls,	Ordinal scale	0-No policy<br>1-Recommend in all public space<br>2-Required in all public space

		supermarkets, and office buildings.		
MS	Mass screening	Record government's policy on PCR testing, which was a key tool used to diagnose and tract infections. During the early stages of the outbreak, PCR testing was primarily conducted for some high-exposure risk individuals. As the outbreak grew, this policy would be extended to some high-risk areas, like neighbourhoods and communities with confirmed cases. Furthermore, the Chinese official also implemented a policy of "universal screening", requiring all residents to be tested.	Ordinal scale	0-No testing<br>1-Testing of people who meet specific criteria (e.g., key careers, key industries, returned from overseas or high-risk areas) or have high risk of exposure<br>2-Testing of people within certain areas (e.g., communities, subdistricts)<br>3-Testing of people within large areas (districts, county, suburbs)<br>4-Testing of anyone within a city (each district is tested in order)<br>5-Testing of anyone within a city (all districts are tested at the same time)
MM	Medicine management	Record management of non-COVID specific medication (e.g., antiviral medication, cough suppressant, fever reducer, etc). Local authorities may require individuals who buy these medications to report to designated hospitals or to be tested for COVID-19. In some cases, the sale of these medications may be restricted to designated hospitals only.	Ordinal scale	0-No measures<br>1-Require registration of usage information<br>2-Require PCR tests for purchasers<br>3-Prohibition on sale
CT	Contact tracing	Record the intensity of contact tracing, a public health measure used to identify and notify	Continuous scale	Number of cases detected in isolation/ Number of

		people who may have been in contact with an infected person. They would also be required to self-quarantine or isolated in designated government shelters.		daily reported cases*0.8

A.2 Processing public-health measure data

In the post-Delta era, China is dedicated to implementing more precise zero-COVID policies to minimise societal impacts. Hence, geographic scope was taken into account in the normalization of measures, calculated as:

$$x_j = (I_j - 1) + \frac{a_j}{max(a_j)}$$

$$x_j^{'} = \frac{x_j}{max(x_j)}$$

Where x_j is the intervention with additional information on geographic scope, I_j indicates the intensity of each measure, a_j is the size of its geographic scope, and $x_j^{'}$ is the normalized measure. Other measures without additional information were normalized by min-max normalization, ranging from 0-1. For PCR-based mass screening, the results were weighted by the frequency of testing performed per week (= total number of tests/7 days). The normalized value, corresponding intensities and additional information including geographic scope and testing frequency for all ordinal variables can be found in Supplementary Table 2. In addition, interventions usually have a lag between implementation and being effective[6,7]. Following the method used in Qiu et al (2022)[7], we tested the relationship between R_t and the intensity of each measure at a 1- to 7-day lag using Spearman's rank correlation coefficient, to determine the minimum time required for each intervention to generate their maximum effect. For each individual

measure, we selected the time lag with the highest correlation coefficient and used it accordingly as the final model input.

Supplementary Table 2. Normalized NPI Lookup - Intensity and Geographic scope. Coding of additional information indicates geographic scope for business premises closures (BPC), public transportation closures (PTC), gathering restrictions (GR), workplace closures (WC), school closures (SC), facial masking (FM), and medicine management (MM), while indicates the total number of tests per week for mass screening (MS).

ID	Normalized intensity	Coding of stringency	Coding of additional information	ID	Normalized intensity	Coding of stringency	Coding of additional information
SO	0.00	0	NA	SC	0.00	0	0
	0.20	1			0.13	1	1
	0.40	2			0.25		2
	0.60	3			0.38		3
	0.80	4			0.50		4
	1.00	5			0.63	2	1
BPC	0.00	0	0		0.75		2
	0.06	1	1		0.88		3
	0.13		2		1.00		4
	0.19		3	FM	0.00	0	NA
	0.25		4		0.50	1	
	0.31	2	1		1.00	2	
	0.38		2	MS	0.00	0	0
	0.44		3		0.03	1	1
	0.50		4		0.06		2
	0.56	3	1		0.09		3
	0.63		2		0.11		4
	0.69		3		0.14		5
	0.75		4		0.17		6
	0.81	4	1		0.20		7
	0.88		2		0.23	2	1
	0.94		3		0.26		2
	1.00		4		0.29		3
PTC	0.00	0	0		0.31		4
	0.06	1	1		0.34		5

	0.13		2
	0.19		3
	0.25		4
	0.31	2	1
	0.38		2
	0.44		3
	0.50		4
	0.56	3	1
	0.63		2
	0.69		3
	0.75		4
	0.81	4	1
	0.88		2
	0.94		3
	1.00		4
**GR**	0.00	0	0
	0.08	1	1
	0.17		2
	0.25		3
	0.33		4
	0.42	2	1
	0.50		2
	0.58		3
	0.67		4
	0.75	3	1
	0.83		2
	0.92		3
	1.00		4
**WC**	0.00	0	0
	0.13	1	1
	0.25		2
	0.38		3
	0.50		4
	0.63	2	1
	0.75		2
	0.88		3
	1.00		4

	0.37		6
	0.40		7
	0.43	3	1
	0.46		2
	0.49		3
	0.51		4
	0.54		5
	0.57		6
	0.60		7
	0.63	4	1
	0.66		2
	0.69		3
	0.71		4
	0.74		5
	0.77		6
	0.80		7
	0.83	5	1
	0.86		2
	0.89		3
	0.91		4
	0.94		5
	0.97		6
	1.00		7
**MM**	0.00	0	0
	0.08	1	1
	0.17		2
	0.25		3
	0.33		4
	0.42	2	1
	0.50		2
	0.58		3
	0.67		4
	0.75	3	1
	0.83		2
	0.92		3
	1.00		4

A.3 Vaccination data

China’s COVID-19 vaccination program officially started in December 2020, after the Chinese government granted conditional approval for vaccines developed by the

Sinopharm[8]. For our study, we collected daily data on vaccination rate, fully vaccination rate and booster rate for each provincial region in mainland China, from the onset of the vaccination program to May 2022 (see supplementary Fig 2). We obtained the data from the website of the National Health Commission of China (http://www.nhc.gov.cn/). Fully vaccination rate refers to the fraction of the total population who have received at least two doses of COVID-19 vaccines. Then, we processed the full vaccination rate into practical vaccination rate which accounts to the efficacy of the used COVID-19 vaccines in China, i.e., Sinovac-CoronaVac and Sinopharm. This allowed us to estimate the proportion of the population that has received at least some level of protection against the infection, even if the effectiveness of vaccination was not as high as expected[9,10]. Next, as the outbreaks occurred at city scale, we further allocated the practical vaccination rate to cities according to the ratio of their population to the corresponding province. Finally, the practical vaccination rate for each city c at day t was defined as:

$$V_t^c = \frac{P_c}{P_p}(FV_t^p \sum_{i=1}^{2} e_f^i + BV_t^p \sum_{i=1}^{2} e_b^i)$$

where e_f^i is the efficacy of full doses against symptomatic COVID-19 for vaccine i, e_b^i is the effect of booster dose against symptomatic COVID-19 for vaccine i from clinical trials (see supplementary Table 3). FV_t^p is the fully vaccination rate of province p at day t, BV_t^p is the booster vaccination rate of province p at day t. P_c is the total population of city c, P_p is the total population of province p where city c belongs to.

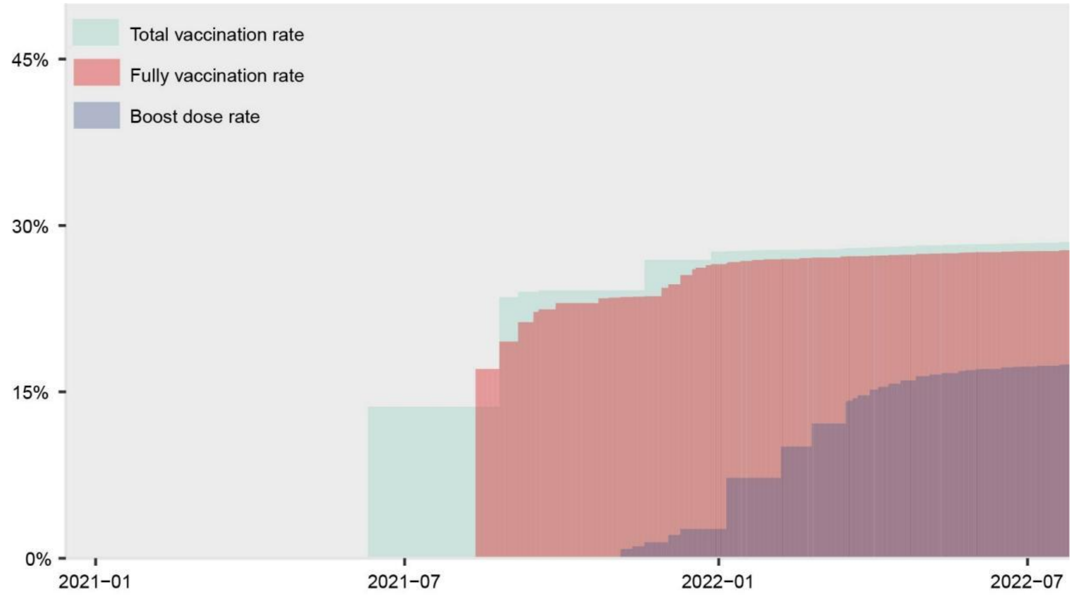

Supplementary Fig 2. Daily vaccination rate in China. Total vaccination rate indicates the fraction of the total population who have received one dose. Fully vaccination rate indicates the fraction of the total population who have received at least two doses of COVID-19 vaccines in China. Boost dose rate indicates the proportion of the fully vaccinated population who have received a booster dose.

Supplementary Table 3. Efficacy of vaccine against symptomatic COVID-19 after full doses (e_f) and booster dose (e_b). The mean value and 95% confidence interval (95% CI) are shown below.

Vaccine	e_f	e_b	Reference
Sinovac-CoronaVac	51%<br>(36-62%)	79%<br>(66-87%)	[11]World Health Organization. *Interim recommendations for use of the inactivated COVID-19 vaccine, CoronaVac, developed by Sinovac: interim guidance, first issued 24 May 2021, updated 21 October 2021, updated 15 March 2022*. https://apps.who.int/iris/handle/10665/352472 (2022).

Sinopharm	50%<br><br>(49-52%)	86%<br><br>(80-91%)	[12]World Health Organization. *Interim recommendations for use of the inactivated COVID-19 vaccine BIBP developed by China National Biotec Group (CNBG), Sinopharm: interim guidance, first issued 7 May 2021, updated 28 October 2021, updated 15 March 2022.* https://apps.who.int/iris/handle/10665/352470 (2022).

A.4 A note on zeroing out China's COVID-19 outbreaks

From December 2019 to March 2020, China invested significant financial and human resources, mainly based on non-pharmaceutical measures, to effectively contain the first wave of COVID-19 in the country [13,14]. However, as COVID-19 spread across countries and cases surged globally, the importation of viruses and new variants presented new challenges. After a successful nationwide containment strategy was adopted in the first wave, China adopted a transitional zero-COVID policy to prevent the resurgence. The core of this policy was to take effective and comprehensive measures to deal with localized COVID-19 cases precisely, to quickly cut off the transmission chain and end outbreaks (to "find one, end one") within one or two maximum incubation periods (i.e., 14 or 28 days [15]). In other words, China aimed to quickly find, control, and cure infected people in each outbreak within a specific geographical region, so as to minimise the impact of COVID-19 on the social and economic development in other regions.

During the Pre-Delta period, this strategy was strict, with limited travel to and from the country, as well as restrictions on domestic movement. The use of masks in public was required with the strictest level, but PCR testing was only regularly carried out on key populations who were close contacts or worked in places with high-risk

exposure risks, with an intensity level below 0.1 daily (Note that NPI intensity ranged from 0 to 1, with 1 indicating the strictest and 0 indicating the least strict measures). However, in the event of a new outbreak, local authorities carried out community PCR mass testing with an intensity above 0.7. The infected residents were hospitalised and isolated, and close contacts were centrally quarantined at facilities such as hotels, along with targeted area lockdowns. Non-essential businesses were required to shut down, with a business premises closure intensity generally above 0.5. In the affected areas of the outbreak, schools were closed down and public transport was suspended with an intensity above 0.6. Working from home was also encouraged to reduce contact in the workplace.

As the SARS-CoV-2 virus continued to evolve, the Chinese government upheld its zero-COVID policy by prioritizing spatially refined prevention and control measures. This was evidenced by a reduction in the intensity of stay-at-home order and contact restrictions. For example, the intensity of stay-at-home order decreased from 0.5 to 0.4 in South Central and Southwest China, while the intensity of business closure decreased from 0.6 to 0.4 in North China. The 10-in-1 pooled testing was used as an alternative to the original individual test, enabling cost-effective mass screening on a large scale. However, the emergence of new, highly transmissible strains of COVID-19 led to the implementation of stricter and more intensive policies. These included more frequent and extensive mass PCR testing in communities/areas that reported local transmission, with the overall intensity of mass screening rising above 0.2.

Since last March 2022, China’s healthcare system has faced increasing pressure due to the spread of Omicron lineages, prompting the relaxation of some regulations. Asymptomatic infections and mild patients no longer need to go to designated hospitals,

but they still need to be isolated at centralized facilities. Lateral flow test kits were available in both offline and online stores, although the results of PCR tests were only accepted to confirm the infection or not. Workplace closures have decreased significantly, with an average intensity dropping to 0 in Northwest China and 0.2 in Southwest China during the Omicron era, compared to 0.6 and 0.4 respectively during the Delta era. In the first 5 months of 2022, over 750,000 cases were detected in mainland China, leading to 2-month-long lockdowns in many cities such as Shanghai. By the end of the study period, China remained intent on zeroing out the infection via ongoing, mass PCR testing and other measures.

B. Estimating the effectiveness of NPIs using Bayesian inference model

B.1 Estimation of instantaneous reproduction number

To estimate the instantaneous reproduction number (R_t) for each outbreak, first, we adjusted the lag from exposure to reporting, to account for the incubation period (i.e., time lag from infection to illness onset or the first positive test) and the reporting delay (i.e. time lag from illness onset or the first positive test to reporting). Specifically, for each case, we inferred a time-lag $t^{'}$, resulting in the infection occurring $t - t^{'}$ days before being reported on day t. This time lag $t^{'}$ is the sum of the incubation period ($t_c^{'}$) and the reporting delay ($t_r^{'}$). The incubation period for each case was sampled from a log-normal distribution with varying means and standard deviations for four main SARS-CoV-2 variants (supplementary Table 4 and Fig 4). The reporting delay was also sampled from a log-normal distribution with a mean of 0.82 days and standard deviation of 0.84 days (see

supplementary Fig 5). Recognizing that large-scale screening following the reporting of index cases might shorten the reporting delay, after the index case was reported, we re-parameterized the onset-to-reporting lag according to a binomial distribution with a mean of 1 day and a standard deviation of 0.3 days (see supplementary Fig 5). The total number of infections on any given day was then counted by aggregating cases by day after adjusting their exposure-to-report delays. We repeated this random sampling process 50 times to get more robustness results. The daily number of infections was calculated as the daily mean value of the 50 samples.

Finally, we estimated R_t using the daily infection numbers and the serial interval of variants, based on the EpiEstim package in R [16]. The number of cases at time t was assumed following the Poisson distribution that defined as:

$$E(I_t) = R_t \sum_{k=1}^{t} I_{t-k} w_k$$

Where I_{t-k} is the incidence at time $t-k$, w_k is the infectivity profile which depends on the serial interval. Serial interval represents the time lag from the illness onset of infectors to the illness onset of infectees. It is a reflection of the natural laws governing the relationship between infectious agents and hosts, depending on the characteristics of the pathogen itself. In practice, we set distinct values of the serial interval for each SARS-CoV-2 variant (see supplementary Table 4 and Fig 3), with a sliding window of 7 days in the estimation. The adjusted reported cases and estimated R_t are available at https://github.com/wxl1379457192/Zeroing_out_emerging_contagions.

Supplementary Table 4. Serial interval and incubation period of each variant.

Serial interval			
Strains	Mean in days	Standard deviation	References
Original	5.80	3.20	[17] Alene, M. *et al.* Serial interval and incubation period of COVID-19: a systematic review and meta-analysis. *BMC Infect Dis* **21**, 257 (2021).
Alpha	3.18	4.36	[18] Geismar, C. *et al.* Household serial interval of COVID-19 and the effect of Variant B.1.1.7: analyses from prospective community cohort study (Virus Watch). *Wellcome Open Res* **6**, 224 (2021).
Delta	2.30	3.40	[19] Zhang, M. *et al.* Transmission Dynamics of an Outbreak of the COVID-19 Delta Variant B.1.617.2 — Guangdong Province, China, May–June 2021. *China CDC Wkly* **3**, 584–586 (2021).
Omicron	2.90	1.60	[20] Song, J. S. *et al.* Serial Intervals and Household Transmission of SARS-CoV-2 Omicron Variant, South Korea, 2021. *Emerg Infect Dis* **28**, 756–759 (2022).
**Incubation period:** $\boldsymbol{t_c^{'} \sim lognormal(x \mid \mu, \sigma)}$			
Strains	μ	σ	References
Original	1.78	0.52	[21] Paul, S. & Lorin, E. Distribution of incubation periods of COVID-19 in the Canadian context. *Sci Rep* **11**, 12569 (2021).
Alpha	1.50	0.46	[22] Tanaka, H. *et al.* Shorter Incubation Period among COVID-19 Cases with the BA.1 Omicron Variant. *Int J Environ Res Public Health* **19**, 6330 (2022).
Delta	1.25	0.34	[23] Ogata, T., Tanaka, H., Irie, F., Hirayama, A. & Takahashi, Y. Shorter Incubation Period among Unvaccinated Delta Variant Coronavirus Disease 2019 Patients in Japan. *Int J Environ Res Public Health* **19**, 1127 (2022).
Omicron	1.02	0.45	[22] Tanaka, H. *et al.* Shorter Incubation Period among COVID-19 Cases with the BA.1 Omicron Variant. *Int J Environ Res Public Health* **19**, 6330 (2022).

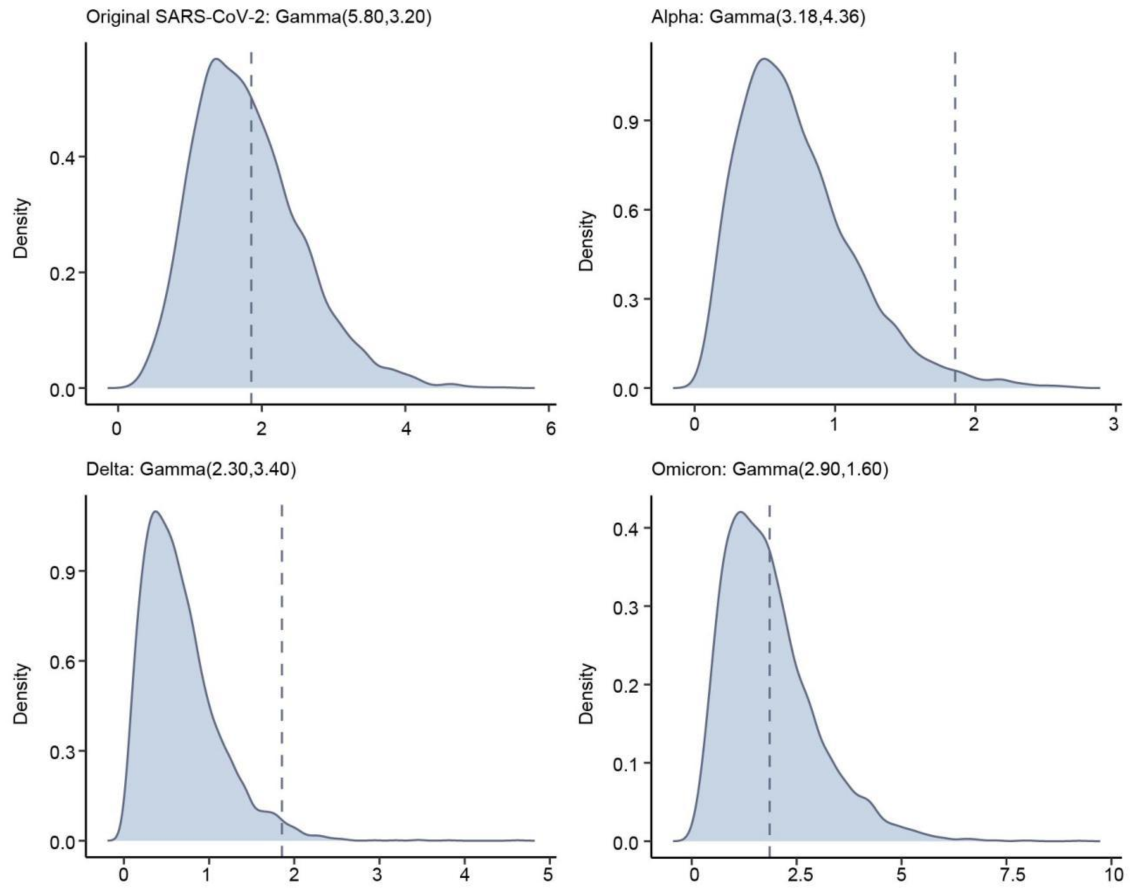

Supplementary Fig 3. The prior distribution of serial intervals for each variant.

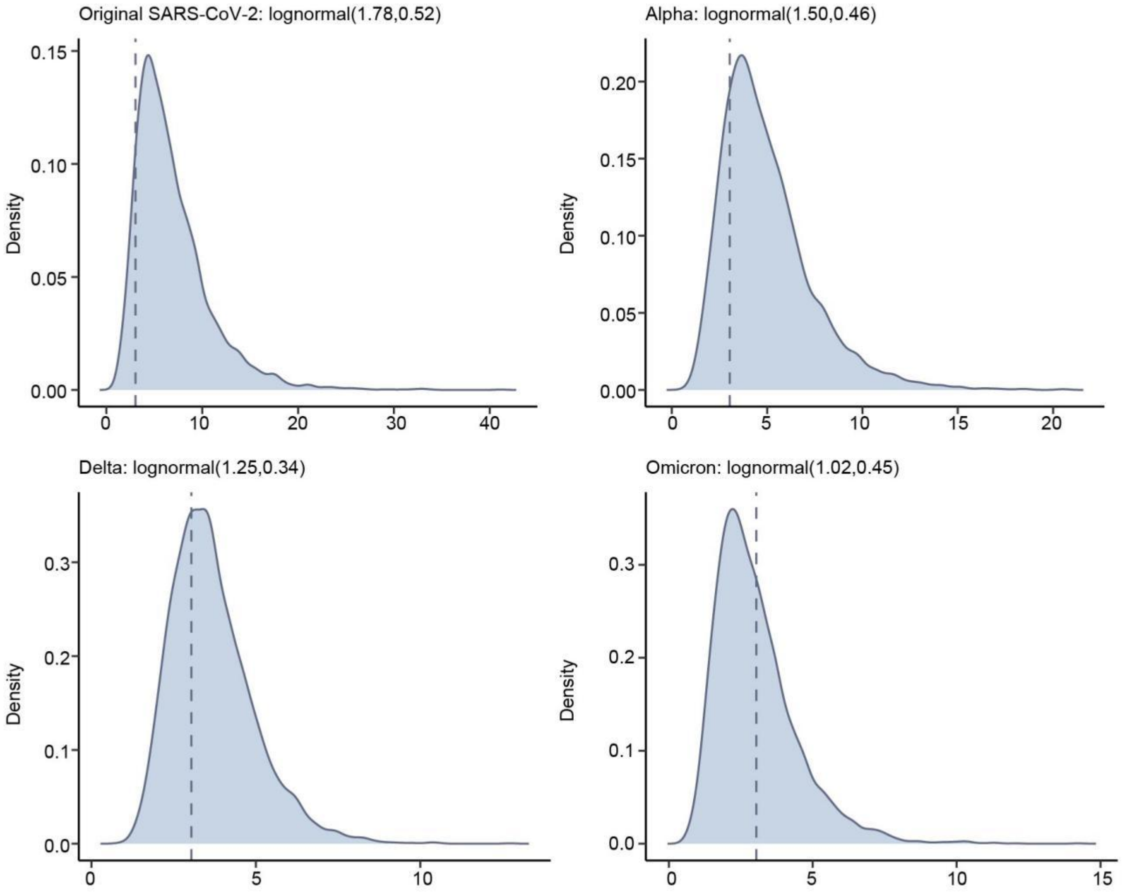

Supplementary Fig 4. The prior distribution of incubation period for each variant.

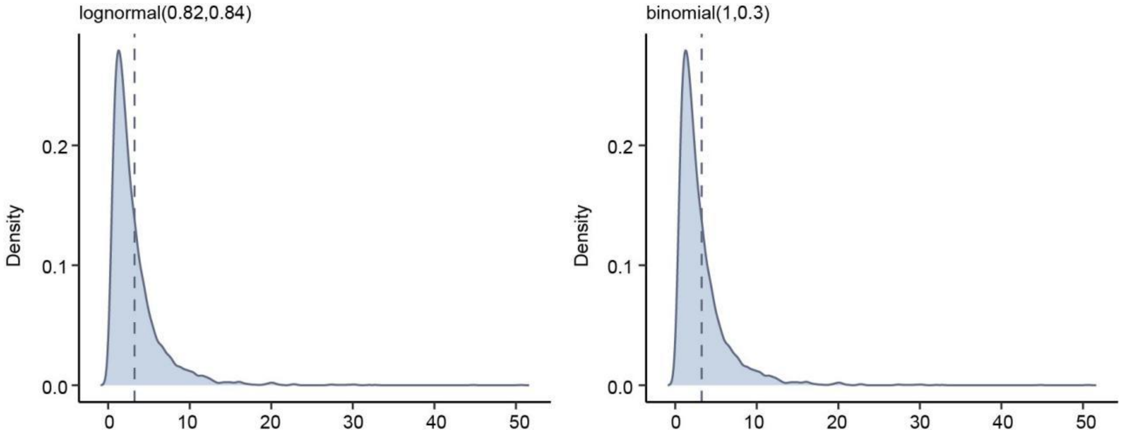

Supplementary Fig 5. The prior distribution of reporting delay (left) and the onset-to-reporting lag (right).

B.2 Basic reproduction number

When estimating the effectiveness of interventions in Bayesian inference models, the basic reproduction number (R_0) was used as a benchmark for comparing the impact of different measures. The effect of a public health measure was evaluated by estimating the reduction in R_0 which results from implementing the intervention. To account for the impact of different factors on R_0, we set R_0 as a hyperparameter obeying the Gamma distribution (supplementary Table 5).

Supplementary Table 5. Basic reproduction number (R_0) of each variant

$R_0 \sim gamma(f, 0.1)$		
Strains	f	References
Original	3.32	[24] Alimohamadi, Y., Taghdir, M. & Sepandi, M. Estimate of the Basic Reproduction Number for COVID-19: A Systematic Review and Meta-analysis. *Journal of Preventive Medicine and Public Health* **53**, 151–157 (2020).
Alpha	4.28	[25] Campbell, F. *et al.* Increased transmissibility and global spread of SARS-CoV-2 variants of concern as at June 2021. *Eurosurveillance* **26**, (2021).
Delta	4.90	[26] Kang, M. *et al.* Transmission dynamics and epidemiological characteristics of SARS-CoV-2 Delta variant infections in Guangdong, China, May to June 2021. *Eurosurveillance* **27**, (2022).
Omicron	9.05	[27] Liu, Y. & Rocklöv, J. The effective reproductive number of the Omicron variant of SARS-CoV-2 is several times relative to Delta. *J Travel Med* **29**, (2022).

B.3 Prior and posterior distribution of effect parameters

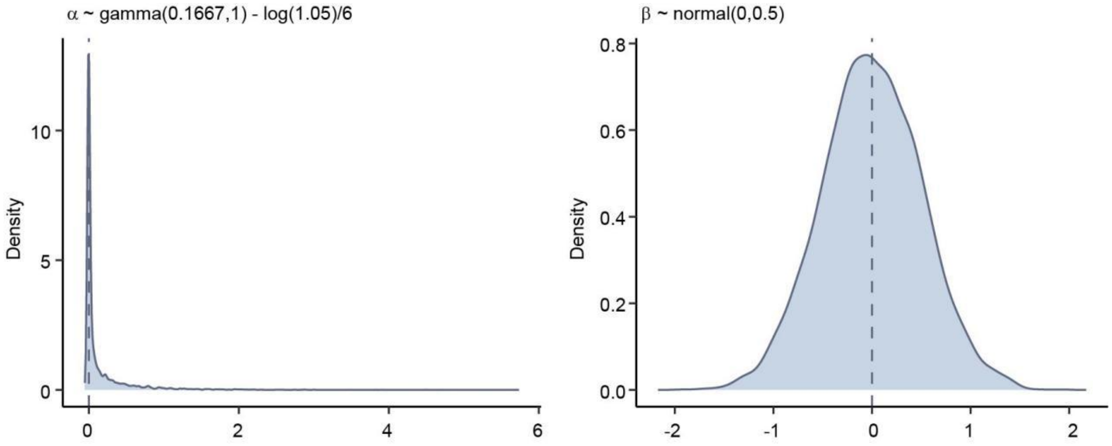

Supplementary Fig 6. The prior distribution of effect parameters of NPIs (left) and the correlation parameter of the control factors (right).

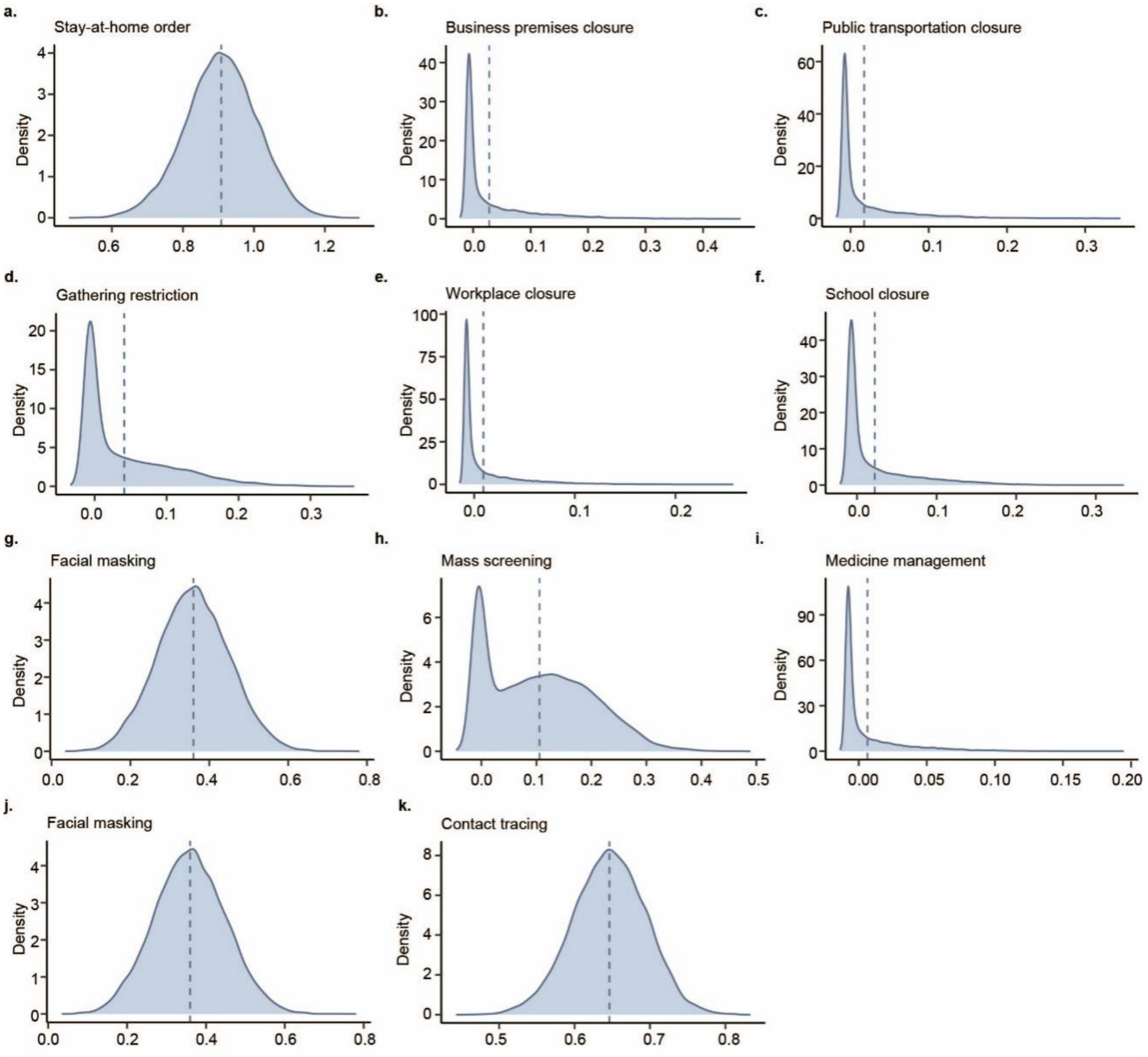

Supplementary Fig 7. The posterior distribution of effect parameters of NPIs (a) stay-at-home order, (b) business premises closure, (c) public transportation closure, (d) gathering restriction, (e) workplace closure, (f) school closure, (g) facial masking, (h) mass screening, (i) medicine management, (j) facial masking, (k) contact tracing.

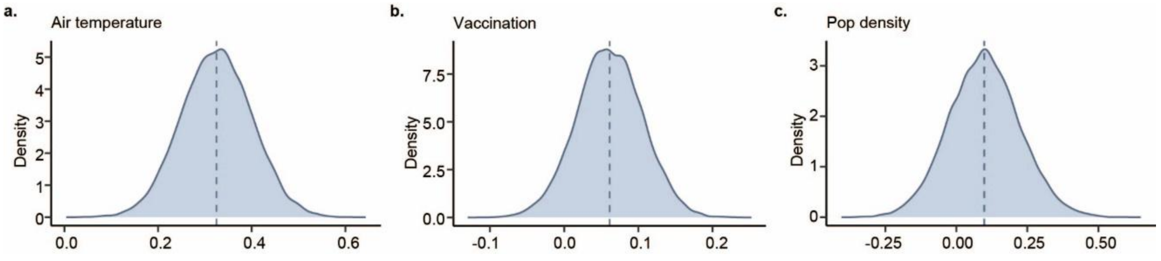

Supplementary Fig 8. The posterior distribution of the correlation parameter of the control factors (a) air temperature, (b) vaccine, (c) population density.

B.4 MCMC convergence

We calibrated our Bayesian inference model with the Markov chain Monte Carlo (MCMC) sampling algorithm. R-hat statistics and relative effective sample size were used to demonstrate the MCMC performance in our model calibration (see supplementary Fig 9).

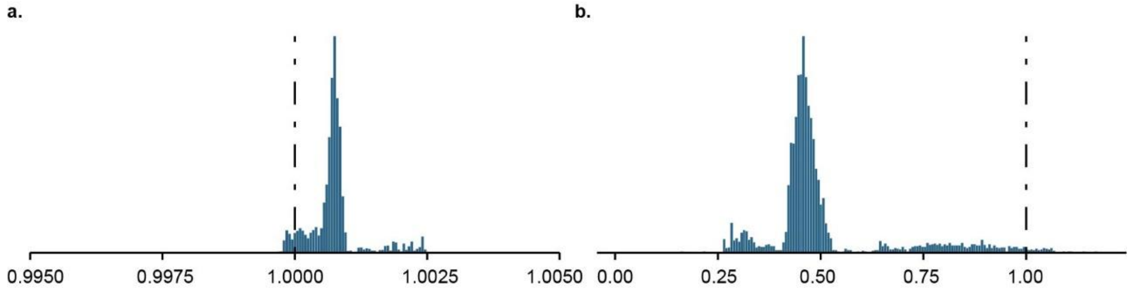

Supplementary Fig 9. The convergence of MCMC in our Bayesian model. (a) R-hat statistic taken from a run using the model with default settings and values for all parameters. Values are close to 1, indicating convergence. (b) Effective sample size taken from a run using the model with default settings and values for all parameters. The value of 1 indicates a perfect decorrelation between samples. Values above (below) 1 indicate that the effective number of samples is higher (lower) than the actual number of samples due to negative (positive) correlation, respectively.

B.5 Leave-one-out cross validation of Bayesian inference model

We used the leave-one-out method to validate our Bayesian inference model. In each run, one outbreak was left out for validation and the rest were used for model training. Using the root mean square error (RMSE) and R-squared, the performance of Bayesian inference model was evaluated by the deviation between the predicted instantaneous reproduction numbers (R_t) and the observed values for all outbreaks. In general, RMSE

ranged from 0 to infinite, with 0 representing perfect predictive power. For example, using this cross-validation approach, we found that the RMSE of the model for 92 Omicron outbreaks ranged from 0.29 to 2.86 (supplementary Fig 10). The average R-squared was 0.68, with an interquartile range of 0.60 - 0.82. The validation results for each Omicron outbreak were summarized in supplementary Table 6.

Supplementary Table 6. Results for the leave-one-out cross validation for Omicron outbreaks.

City code	City name	Start date	RMSE	R squared
110000	Beijing	2020/6/11	1.88	0.51
		2020/12/23	1.18	0.7
		2022/1/15	0.76	0.86
		2022/3/7	0.92	0.67
		2022/4/20	1.20	0.59
120000	Tianjin	2022/1/8	1.32	0.64
		2022/3/7	1.19	0.75
		2022/5/12	1.01	0.86
130100	Shijiazhuang	2021/1/2	2.67	0.40
		2021/10/23	0.76	0.96
130200	Tangshan	2022/3/19	1.75	0.66
		2022/4/18	1.73	0.24
130400	Handan	2022/4/1	1.18	0.70
130600	Baoding	2022/3/28	1.34	0.95
130900	Cangzhou	2022/3/8	1.10	0.74
131000	Langfang	2022/3/9	1.51	0.84
140100	Langfang	2022/4/3	1.05	0.65
150100	Hohhot	2022/2/15	0.69	0.75

150700	Hulunbuir	2021/11/27	0.58	0.59
152900	Alxa	2021/10/19	0.55	0.87
210100	Shenyang	2022/3/6	0.64	0.77
210200	Dalian	2020/7/22	2.17	0.41
		2020/12/15	0.83	0.78
		2021/11/4	0.64	0.76
		2022/3/14	1.13	0.92
210600	Dandong	2022/4/24	1.54	0.60
		2022/5/24	1.62	0.17
210800	Yingkou	2022/3/13	1.03	0.89
211400	Huludao	2022/2/8	0.53	0.74
220100	Changchun	2021/1/11	0.89	0.73
		2022/3/4	0.90	0.97
220200	Jilin	2022/3/1	0.71	0.88
220300	Siping	2022/3/10	0.88	0.83
220500	Tonghua	2021/1/12	1.63	0.64
220600	Baishan	2022/5/13	1.10	0.45
220800	Baicheng	2022/3/29	1.11	0.82
222400	Yanbian	2022/3/2	0.6	0.76
		2022/4/15	0.97	0.63
230100	Harbin	2020/4/9	1.20	0.65
		2021/9/21	0.63	0.81
		2022/3/8	0.96	0.89
		2022/4/13	1.66	0.00
230800	Jiamusi	2022/3/27	0.89	0.77
231000	Mudanjiang	2022/1/25	0.46	0.62
231100	Heihe	2021/10/27	0.58	0.86
310000	Shanghai	2022/2/26	0.92	0.8
320100	Nanjing	2021/7/20	0.53	0.78

		2022/3/10	0.79	0.73
320200	Wuxi	2022/3/29	0.57	0.81
320300	Xuzhou	2022/3/26	1.13	0.74
		2022/4/16	1.71	0.21
320400	Changzhou	2022/3/13	1.33	0.75
320500	Suzhou	2022/2/10	0.48	0.84
		2022/3/9	1.00	0.11
320600	Nantong	2022/3/21	0.85	0.74
320700	Lianyungang	2022/3/5	0.98	0.89
321000	Yangzhou	2021/7/28	0.59	0.86
321300	Suqian	2022/3/27	1.59	0.75
330100	Hangzhou	2022/1/26	1.59	0.39
		2022/3/5	0.83	0.53
330200	Ningbo	2021/12/6	0.66	0.77
		2022/3/28	0.73	0.58
330400	Jiaxing	2022/3/11	0.75	0.55
330600	Shaoxing	2021/12/7	0.71	0.81
330700	Jinhua	2022/3/26	0.95	0.64
330800	Quzhou	2022/3/7	0.83	0.82
340200	Wuhu	2022/3/24	0.93	0.74
340400	Huainan	2022/3/27	0.99	0.84
340700	Tongling	2022/3/14	0.92	0.85
341200	Fuyang	2022/3/27	1.17	0.68
341500	Lu'an	2022/4/2	1.45	0.77
350200	Xiamen	2021/9/12	0.66	0.81
350300	Putian	2021/9/10	0.76	0.82
		2022/3/17	1.12	0.63
350500	Quanzhou	2022/3/13	1.3	0.92
350900	Ningde	2022/3/30	0.38	0.95

360100	Nanchang	2022/3/13	0.95	0.83
360900	Yichun	2022/5/7	1.11	0.6
361100	Shangrao	2021/10/30	0.55	0.69
		2022/4/20	1.70	0.75
370100	Jinan	2022/3/29	0.58	0.68
370200	Qingdao	2022/3/1	1.34	0.92
370300	Zibo	2022/3/8	0.94	0.77
370600	Yantai	2022/4/22	1.53	0.26
371000	Weihai	2022/3/7	0.92	0.84
371300	Linyi	2022/3/15	0.77	0.46
371400	Dezhou	2022/3/10	0.98	0.70
371600	Binzhou	2022/3/11	1.41	0.88
410100	Zhengzhou	2021/7/31	0.43	0.81
		2021/11/3	0.83	0.81
		2022/1/3	0.66	0.97
		2022/4/8	0.93	0.80
410500	Anyang	2022/1/8	1.09	0.79
		2022/4/8	0.41	0.73
411000	Xuchang	2022/1/2	0.63	0.83
		2022/5/3	1.21	0.58
411600	Zhoukou	2022/3/22	0.85	0.76
		2022/5/1	1.32	0.65
420100	Wuhan	2021/8/2	0.52	0.78
		2022/4/1	0.57	0.90
420700	Ezhou	2022/3/27	1.03	0.78
430800	Zhangjiajie	2021/7/29	0.59	0.81
440100	Guangzhou	2021/5/23	0.3	0.83
		2022/3/30	1.17	0.78
		2022/4/28	1.55	0.05

440300	Shenzhen	2022/2/12	1.06	0.62
440800	Zhanjiang	2022/5/6	1.09	0.53
441900	Dongguan	2022/2/25	0.9	0.7
450600	Fangchenggang	2022/2/23	0.85	0.5
		2022/4/5	1.76	0.57
450700	Qinzhou	2022/3/13	1.08	0.72
451000	Baise	2022/2/5	1.43	0.59
451400	Chongzuo	2022/3/11	1.03	0.64
460200	Sanya	2022/3/31	1.38	0.52
500000	Chongqing	2022/3/12	0.85	0.75
510100	Chengdu	2022/3/28	0.96	0.85
511600	Guangan	2022/5/9	1.27	0.95
530900	Lincang	2022/2/23	0.91	0.56
533100	Dehong	2021/3/30	1.84	0.53
		2021/7/4	0.64	0.40
		2021/10/1	0.7	0.15
		2022/2/16	0.55	0.53
610100	Xi'an	2021/12/12	0.59	0.84
		2022/3/5	0.93	0.67
		2022/4/2	1.27	0.79
620100	Lanzhou	2021/10/19	0.42	0.52
		2022/3/7	0.81	0.74
630100	Xining	2022/4/3	0.65	0.60
650100	Wulumuqi	2020/7/15	2.04	0.60
		2022/4/27	1.47	0.58
653100	Kashgar	2020/10/24	2.86	0.33

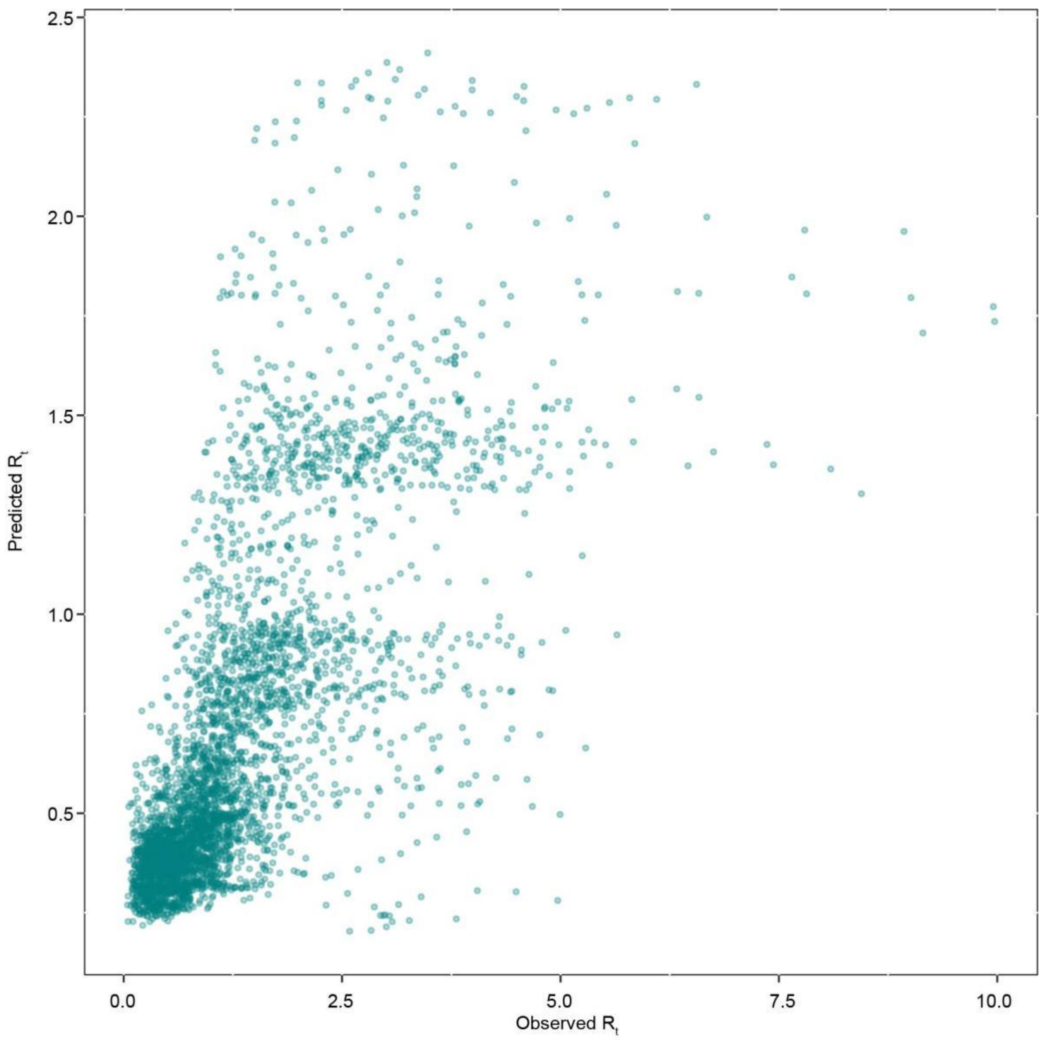

Supplementary Fig 10. The results for leave-one-out validation of modelling 92 Omicron outbreaks. The overall R squared is 0.50.

B.6 Impact of temperature and vaccination

Due to the diversity among study regions, we considered air temperature (2 meters above the surface), practical vaccination rate and population density as city-specific confounders in the estimation of intervention effects. Their effects were visualized in Supplementary Fig 11. Overall, both the vaccination (18%, 95%CI 6-30%) and air temperature (23%, 95%CI 15-31%) showed a positive effect on containing the spread,

with air temperature playing a more significant role. The results did not provide substantial evidence indicating a significant effect of population density on virus transmission.

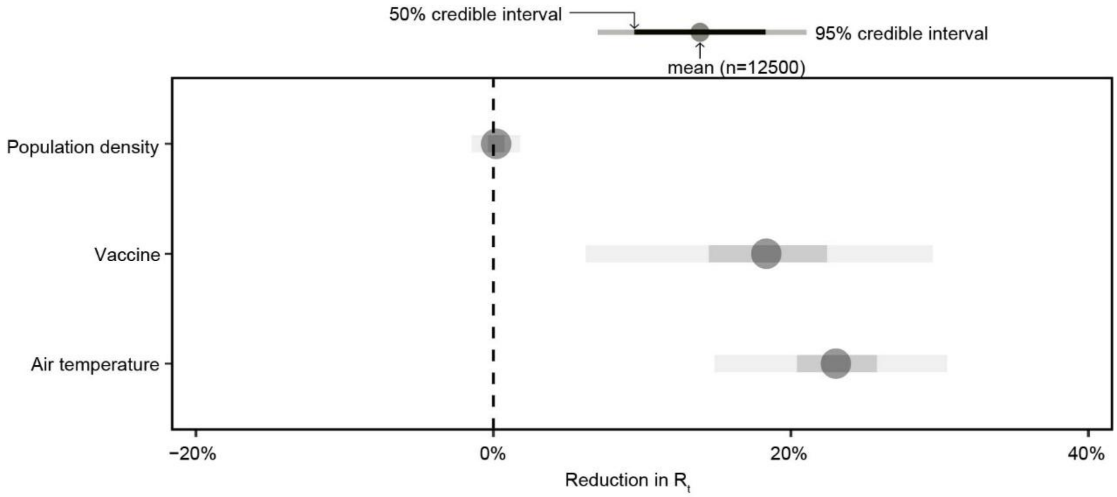

Supplementary Fig 11. The relative effects of air temperature and vaccination on reducing the transmission of COVID-19 under Pre-Delta, Delta and Omicron era. The minimum, maximum, median, 25th percentile (Q1) and 75th percentile (Q3) of estimates on the reduction in R_t (%) are presented to illustrate more details about the observed overall reduction in propagation.

B.7 Effect of China's zero-COVID policy within each group

The 131 outbreaks were classified into four groups against each variant using the geodetector model [28], a geospatial analysing tool that helps identify spatial patterns of events and their interactions with environment factors. Due to the stratified heterogeneity of the infection rate across outbreaks (supplementary Fig 12), these outbreaks were divided into four groups: small-scale outbreaks lasting for a short period of time (Group 1), large-scale outbreaks lasting for a short period of time (Group 2), small outbreaks of

longer duration (Group 3) and large-scale outbreaks of longer duration (Group 4). We started by analysing the variability in policy efficacy among outbreaks of varying scales as shown in supplementary Fig 13. The effectiveness of social distancing measures increased with the scale of outbreaks, reaching 31% (95%CI 22-39%) in small-scale, short-duration outbreaks (Group 1) and 69% (95%CI 52-80%) in larger, long-duration outbreaks (Group 4). Facial masking was found to be effective in reducing infection rates by up to 67% (95%CI 32-86%) in long-duration outbreaks. Contact tracing was most effective in groups with fewer cases, such as Group 1 (32%, 95%CI 28-36%) and Group 2 (9%, 95%CI -1-28%). To investigate the role of each NPI in outbreaks of varying scales and strains, we also estimated their effects within groups under each variant (supplementary Fig 14). Our results indicate that PCR mass screening was relatively more effective in fighting against COVID-19 in groups with sustainable transmission compared to the outbreaks that were rapidly curbed.

In the Pre-Delta era, social distancing measures had the highest contribution to transmission reduction, especially in Group 2 with long duration and low transmission (91%, 95% CI 77-98%). Facial masking showed its excellent transmission-blocking ability in Group 3 (70%, 95% CI 29-88%), which has a long duration and small infection size. The effectiveness of PCR screening increased with the severity of the outbreak, with 4% (95%CI 0-22%), 7% (95%CI 0-38%), 23% (95%CI 0-47%), 28% (95%CI -1-90%) for each group. Contact tracing has demonstrated exceptional performance (13%, 95%CI 0-34%) in swiftly containing the spread of the pandemic, like Group 1.

During the Delta period, the effectiveness of social distancing measures in containing pandemics began to decline. For example, the effect of social distancing

measures decreased from 49% (95%CI 30-66%) to 23% (95%CI 0-46%) in Group 1, 91% (95%CI 77-98%%) to 40% (95%CI -1-92%) in Group 2, 37% (95%CI 0-65%) to 10% (95%CI -2-36%) in Group 3 and 77% (95%CI 14-95%) to 29% (95%CI -1-65%) in Group 4. Facial masking had a more important role in reducing transmission, with an increase of 18%, 42%, 9%, 20% in Group1, Group 2, Group 3, Group 4, respectively. PCR testing remained effective when the infection persisted over a long period (over 6%), otherwise when the pandemic was rapidly controlled (about 3%). Contact tracing had a significant effect in Group 2 (20%, 95%CI -1-66%).

For Omicron variants, facial masking became the most effective NPI in long-term outbreaks, where 83% (95%CI 67-92%) for Group 2. PCR screening is only effective in wide-spread transmission, i.e. Group 4 (6%, 95%CI -1-31%), while social distancing measures made notable contributions in completely eliminating the virus from Group 4 (60%, 95%CI 26-78%). Contact tracing outperformed in this era, especially in outbreaks with only a short stay. And its effectiveness was shown as 36% (95%CI 31-40%) in Group 1, 11% (95%CI -1-30%) in Group 2 and 2% (95%CI 0-16%) in Group 4.

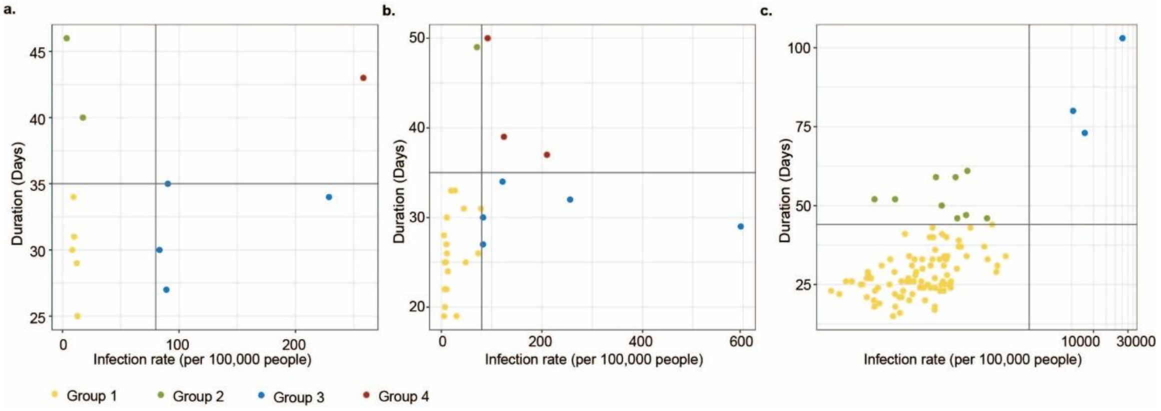

Supplementary Fig 12. Distribution of the 131 outbreaks in terms of infection rates and durations under (a) Pre-Delta era, (b) Delta era, (c) Omicron era. The grey lines represent the infection rate and duration thresholds.

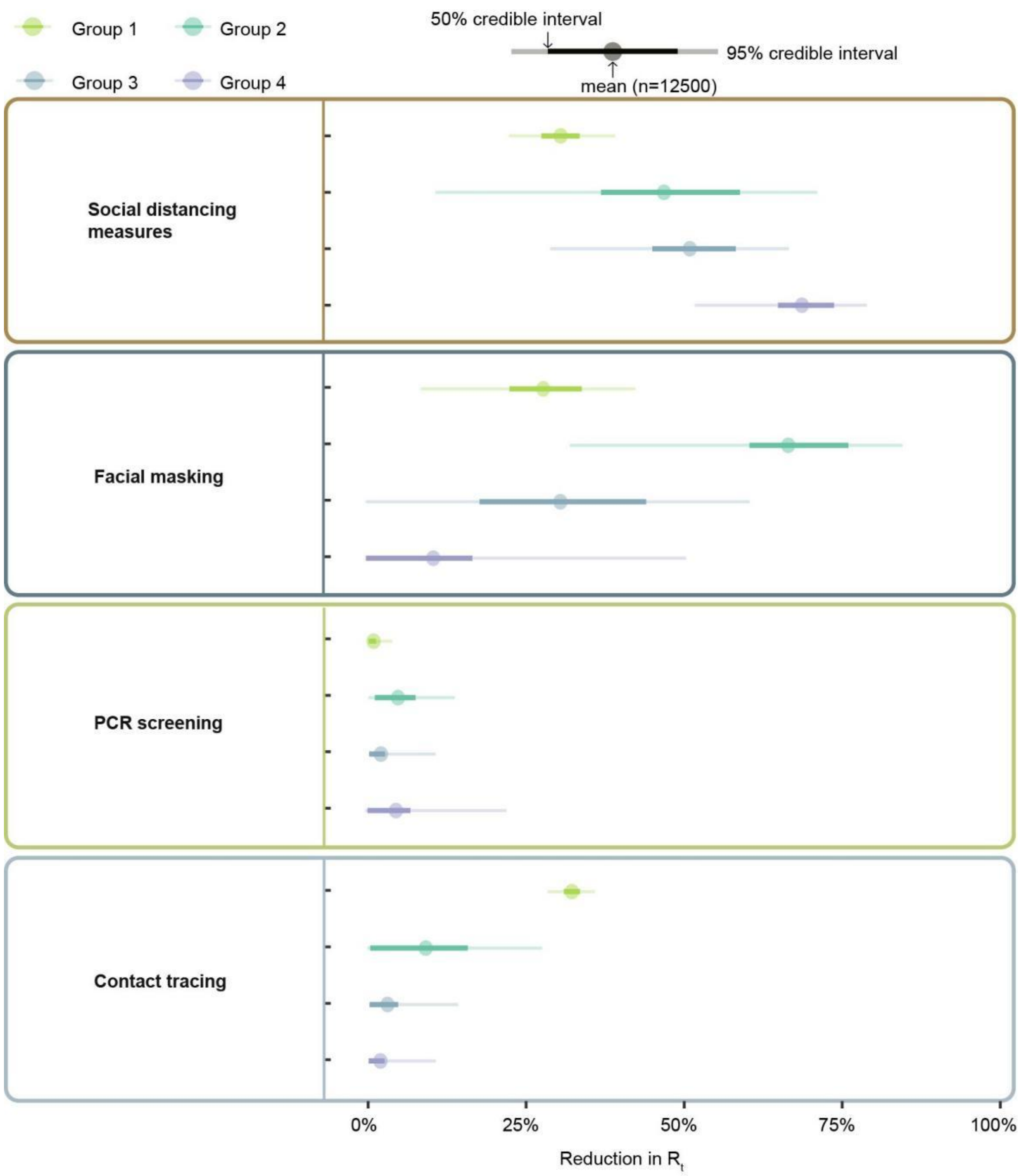

Supplementary Fig 13. Effectiveness of NPIs on reducing the transmission of COVID-19 within each group. PCR screening shows the synergistic effect of the mass screening, the medicine management and the contact control. Social distancing measures

show the synergistic effect of stay-at-home order, business premises closure, public transportation closure, gathering restriction, workplace closure and school closure. The effect estimates are computed by the coefficients of each individual NPI through $1 - exp\,(-\sum_i^n \alpha_{i,v}\overline{X_{i,t,v}})$, where $\overline{X_{i,t,v}}$ is the median value of the NPI implementation intensity and the coefficients ($\alpha_{i,v}$) is derived by fitting the data with the default settings within each group for each variant. The minimum, maximum, median, 25th percentile (Q1) and 75th percentile (Q3) of estimates on the reduction in R_t (%) are presented to illustrate more details about the observed overall reduction in propagation.

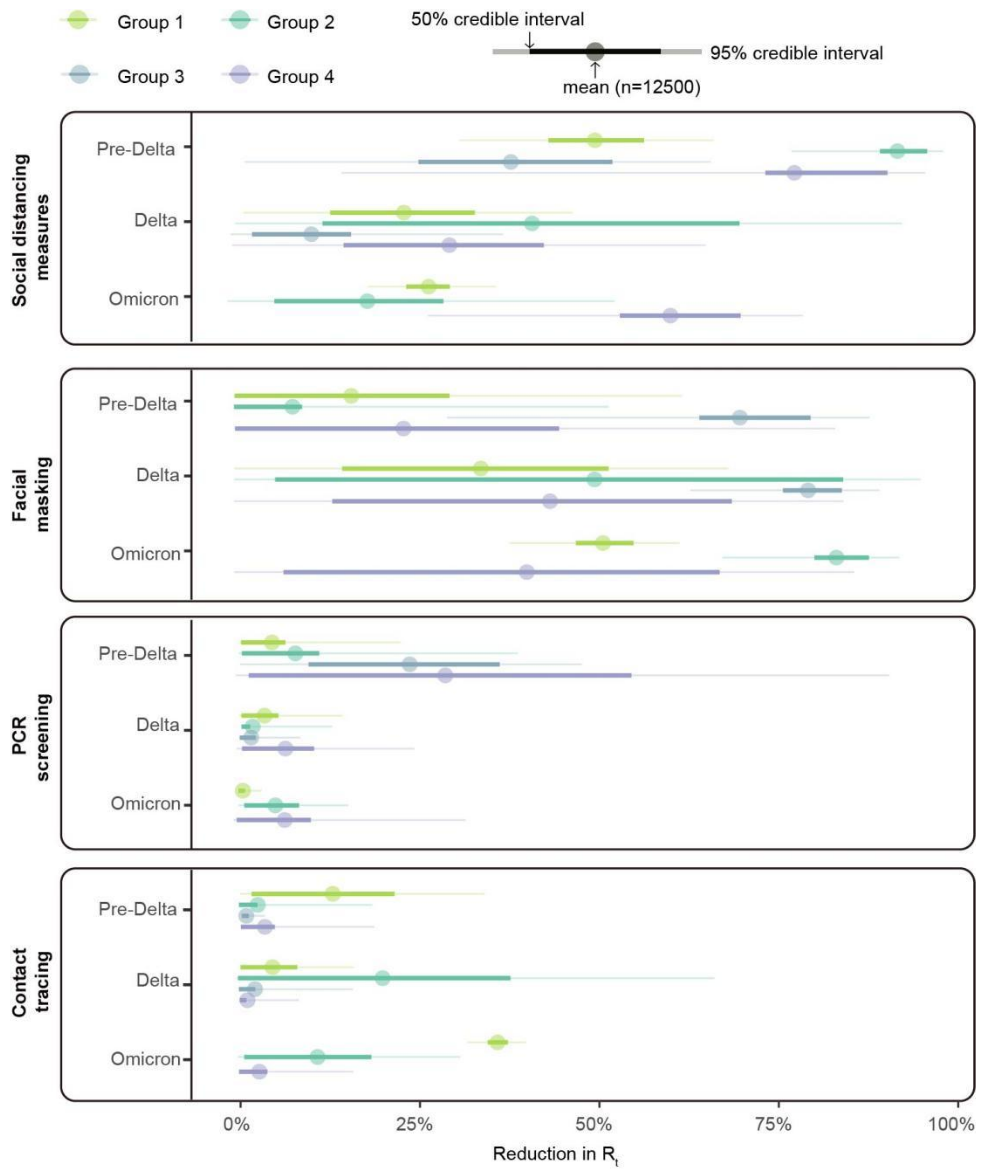

Supplementary Fig 14. Effectiveness of NPIs on reducing the transmission of COVID-19 under Pre-Delta, Delta and Omicron era within each group. PCR screening shows the synergistic effect of the mass screening, the medicine management and the contact control. Social distancing measures show the synergistic effect of stay-at-home order, business premises closure, public transportation closure, gathering restriction, workplace closure and school closure. The effect estimates are computed by the coefficients of each individual NPI through $1 - exp\,(-\sum_i^n \alpha_{i,v}\overline{X_{i,t,v}})$, where $\overline{X_{i,t,v}}$ is the

median value of the NPI implementation intensity and the coefficients ($\alpha_{i,v}$) is derived by fitting the data with the default model settings under different variant eras. The minimum, maximum, median, 25th percentile (Q1) and 75th percentile (Q3) of estimates on the reduction in R_t (%) are presented to illustrate more details about the observed overall reduction in propagation.

C. ISEIRV model

C.1 Calibration of ISEIRV model

A Bayesian optimisation framework was developed to optimize quantization hyperparameters in ISEIRV models, including 1) the baseline of transmission rate b_0, 2) the baseline of recovery period r_0, 3) the coefficients, b_1, r_1, r_2, and r_3, 4) the initial exposed population $E(0)$, and 5) the initial infectious population $I(0)$. The initial prior distributions of the unknown parameters are listed in Supplementary Table 7. We set the initial expect value of r_0 as 7, 6 and 5 for the pre-Delta, Delta and Omicron era, respectively. The upper bound for the prior distribution of r_3 is outbreak specific as the ongoing days of the outbreak.

First, these parameters were simultaneously sampled 2000 times from their prior distributions. Second, the daily new infections were stimulated by the ISEIRV model using each of 2000 sets of sampled parameters, and the mean squared error between the simulated daily number of cases and the reported number was calculated then. Third, the 2000 samples of the parameter sets were ranked by the corresponding mean squared error, where 400 samples of the parameter set with the smallest mean squared error were used to fit a Gaussian distribution as the new prior distributions. The above procedure was

iterated 15 times, and the last updated prior distributions were determined as the final posterior distributions of the corresponding underestimated parameters.

Supplementary Table 7. The priori distributions of the unknown parameters.

Parameters	Distribution
b_0	$normal(\frac{f}{r_0}, 1)$
r_0	$normal(r_0, 1)$
b_1	$uniform(0, 3)$
r_1	$uniform(0, 3)$
r_2	$uniform(0, 1)$
r_3	$uniform(0, length(cases)$
$E(0)$	$uniform(5, 15)$
$I(0)$	$uniform(1, 5)$

C.2 Model validation against prediction accuracy

We split each outbreak into two successive time windows, the training set and the validation set, to test the predictive accuracy of the ISEIRV model. The initial 85% days in an outbreak were used to train the model and fit the parameters, and the following days were used to validate the performance of the trained model. We set 5 days as the shortest of the validation input, which means it will be extended to the last 5 days of an outbreak when the validation set is shorter than 5 days. In terms of the number of cumulative cases and daily new cases, the normalized root means square error (NRMSE) and r squared (R^2) were employed to estimate the performance of models under real-world scenarios. Although the simulation error of 100 infected individuals differed more from each other

than the error of 10 infected individuals in absolute values, this difference was relatively small when compared to an outbreak with tens of thousands of cases. In this case, NRMSE can facilitate the comparison of datasets or models with different scales, by normalizing the root mean square error (RMSE). In this study, we used the mean value of the daily reported number of cases that has been adjusted for the infection-to-report delay to normalize the RMSE for each outbreak.

C.3 Robustness of ISEIRV model

The ISEIRV model was constructed for each of the 131 outbreaks in mainland China, occurring from April 1, 2020 to May 31, 2022. We validated the model against the observed epidemic timeline of each outbreak, confirming that the adopted parameter scheme was acceptable. The observed cumulative cases were also compared with the simulated data to indicate the robustness of our model, as shown in supplementary Fig 15. The adjusted R^2 is 0.47, highlighting the reasonable parameters assumption of our model. In addition, the accuracy performance of the ISEIRV model in simulating the daily infected individuals was also tested on the basis of the validation set for each outbreak. Under the pre-Delta era, the simulations were the most robust, with NRMSE ranging from 0.09 to 0.41 and mean R^2 of 0.91. In the period of Delta and Omicron, the simulated daily cases showed some deviations from the observed values in some outbreaks, but most of them still reproduced the epidemic progressions ideally. Supplementary Fig 13b summarized the details of point-by-point validation results of the ISEIRV model. The mean NRMSE was 0.20 in the Delta period and 0.17 for Omicron outbreaks, respectively, while the mean R^2 was 0.89 in the Delta era and 0.88 in the Omicron era. This further

confirmed that the ISEIR model proposed in our study has good performance in simulating COVID-19 outbreaks. As the robustness of ISEIRV model has been validated, we confirmed that the adopted parameter scheme was acceptable, as shown in supplementary Table 8.

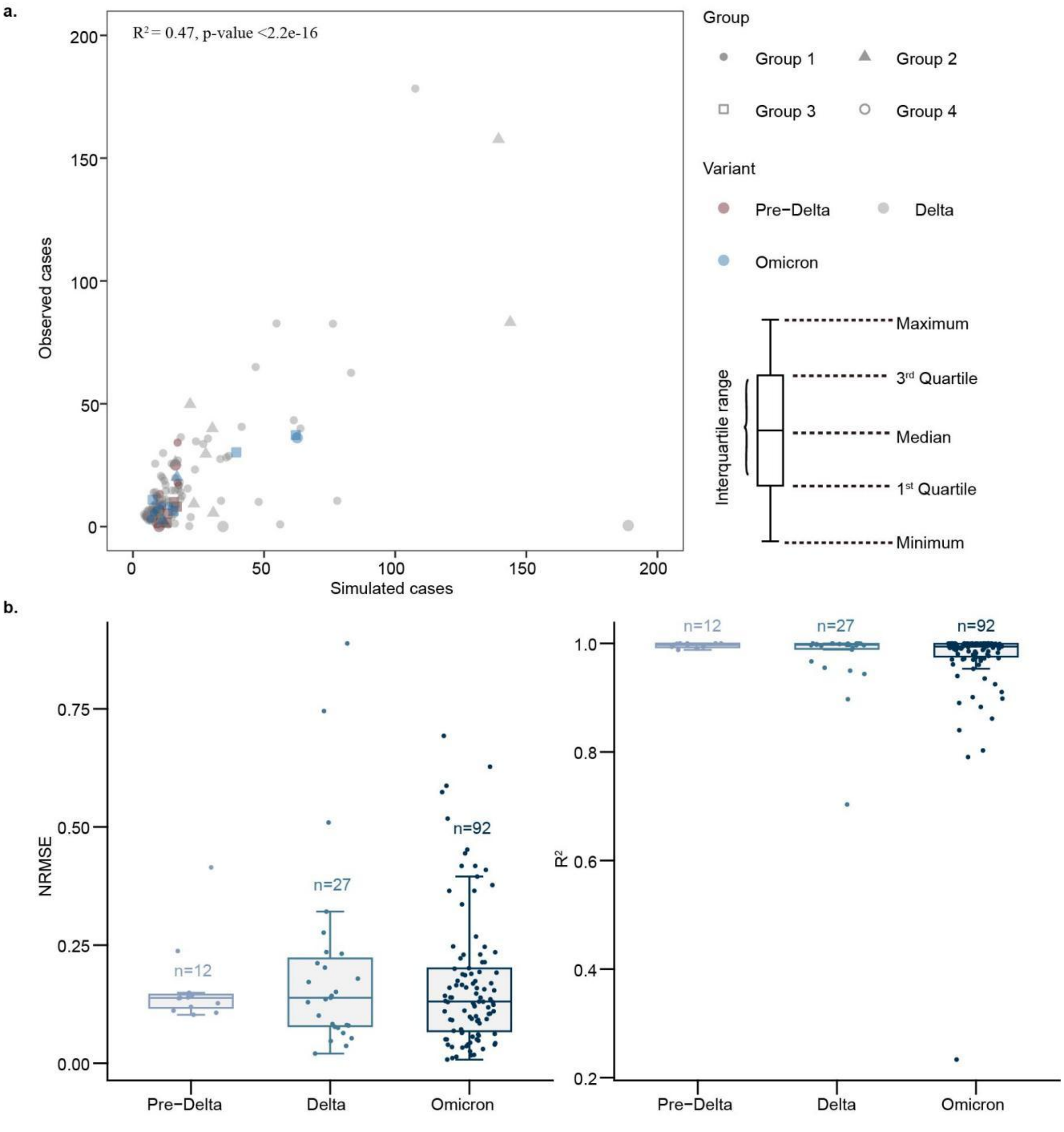

Supplementary Fig 15. The performance of the ISEIRV model in simulating cumulative infections (a) and daily infections (b). The scatterplot (a) compared the

simulated cumulative infections versus the observed cumulative infections for 131 outbreaks in each group for each variant. P-values are produced by two-sided Wilcoxon test. The boxplot (b) summarized the NRMSE and R^2 of the simulated daily infections found from COVID-19 propagation modelling during the Per-Delta, Delta and Omicron periods. The scatter in each box plot represented the point-to-point simulated precision of each outbreak.

Supplementary Table 8. Estimated parameter values of the Intervention-SEIRV model. b_0 indicates the baseline of the transmission rate. r_0 indicates the baseline of recovery period. b_1 is the effect of contact related NPIs on reducing individual-level contact frequency. r_1 is the effect of the infectious detection related NPIs on improving detection rate. r_2 and r_3 are coefficients to portray the policy lag. $E(0)$ indicates the initial exposed population. $I(0)$ indicates the initial infectious population.

Variant	City code	City name	Start date	b_0	b_1	r_0	r_1	r_2	r_3	$E(0)$	$I(0)$
Pre-Delta	230100	Harbin	20200409	0.64	2.38	3.75	4.06	1.13	5.31	10.03	6.45
	110000	Beijing	20200611	1.09	1.56	4.06	3.55	1.04	6.01	20.67	6.80
	650100	Wulumuqi	20200715	1.31	2.30	7.89	0.65	0.46	10.69	14.07	4.28
	210200	Dalian	20200722	0.90	1.08	4.39	3.99	1.10	4.07	14.19	6.94
	653100	Kashgar	20201024	1.26	1.39	4.48	3.81	0.98	5.93	20.35	6.52
	210200	Dalian	20201215	0.68	2.53	3.57	2.41	1.14	4.25	11.04	6.87
	110000	Beijing	20201223	0.62	1.17	7.18	1.02	0.77	38.66	5.86	1.02
	130100	Shijiazhuang	20210102	1.41	2.32	5.20	2.96	0.96	7.88	18.97	6.19
	220100	Changchun	20210111	0.81	1.12	7.01	0.50	0.27	9.90	11.20	4.68
	220500	Tonghua	20210112	0.99	1.86	5.22	2.45	0.93	5.79	16.45	5.61

	533100	Dehong	20210330	0.78	2.27	3.46	3.37	1.14	5.30	16.61	7.86
	440100	Guangzhou	20210523	1.17	2.50	4.47	0.33	1.10	4.20	16.75	7.08
Delta	533100	Dehong	20210704	0.79	2.00	4.91	2.47	0.69	0.95	13.97	6.64
	320100	Nanjing	20210720	1.07	2.60	2.55	4.98	1.11	6.20	20.57	7.22
	321000	Yangzhou	20210728	1.75	2.22	5.76	2.36	0.39	8.06	14.37	4.70
	430800	Zhangjiajie	20210729	0.96	1.20	6.64	0.75	0.79	17.88	6.55	3.04
	410100	Zhengzhou	20210731	0.72	2.79	2.67	4.09	1.13	6.52	16.39	7.26
	420100	Wuhan	20210802	0.92	2.51	3.49	2.82	1.07	4.39	9.30	6.05
	350300	Putian	20210910	1.93	2.24	2.59	5.12	1.13	4.84	20.64	7.24
	350200	Xiamen	20210912	2.23	2.26	4.16	2.80	0.92	4.01	17.55	6.41
	230100	Harbin	20210921	1.34	2.35	3.61	3.19	1.02	3.82	10.85	6.52
	533100	Dehong	20211001	1.48	1.44	6.47	1.39	0.78	45.19	5.45	3.84
	152900	Alxa	20211019	1.05	1.23	6.30	0.29	0.49	2.09	8.66	5.37
	620100	Lanzhou	20211019	1.87	2.39	5.63	2.33	0.76	5.93	10.82	4.97
	130100	Shijiazhuang	20211023	2.11	1.62	3.19	4.33	1.06	2.76	9.23	6.22
	231100	Heihe	20211027	2.14	2.11	4.06	2.29	0.99	3.72	17.75	6.13
	361100	Shangrao	20211030	1.10	1.13	7.22	0.81	0.08	10.49	7.97	4.25
	410100	Zhengzhou	20211103	1.79	1.77	4.37	3.40	0.95	4.40	9.87	5.80
	210200	Dalian	20211104	3.53	0.01	3.47	4.11	1.09	4.11	13.45	7.22
	150700	Hulunbuir	20211127	2.25	1.90	2.74	3.96	1.07	6.17	20.75	7.31
	330200	Ningbo	20211206	2.00	2.39	3.68	2.47	0.99	2.90	12.76	6.66
	330600	Shaoxing	20211207	2.61	1.69	4.36	2.74	0.89	5.20	18.34	6.27
	610100	Xi'an	20211212	2.49	0.19	3.40	3.88	1.13	2.62	19.75	7.58
	411000	Xuchang	20220102	2.37	1.49	5.80	1.30	0.69	4.47	15.54	5.64
	410100	Zhengzhou	20220103	2.26	1.55	3.69	3.82	0.97	4.08	16.08	7.01
	110000	Beijing	20220115	2.65	1.35	5.55	1.63	0.89	2.78	13.21	6.27

	231000	Mudanjiang	20220125	1.89	1.25	3.76	3.26	0.95	2.97	7.56	5.49
	211400	Huludao	20220208	1.69	1.70	6.29	0.09	0.36	11.39	16.29	6.09
	150100	Hohhot	20220215	2.82	1.13	4.59	1.58	0.93	5.05	20.21	6.96
Omicron	120000	Tianjin	20220108	3.03	1.42	2.58	3.34	1.04	5.30	18.36	6.34
	410500	Anyang	20220108	3.00	2.45	3.30	2.90	0.80	5.65	17.12	6.16
	330100	Hangzhou	20220126	3.04	0.83	2.37	3.93	1.13	3.53	13.67	6.71
	451000	Baise	20220205	2.97	2.51	1.84	3.74	1.20	4.93	20.34	7.42
	320500	Suzhou	20220210	2.65	2.52	2.31	4.42	1.13	4.26	14.50	7.12
	440300	Shenzhen	20220212	3.07	0.40	3.39	2.91	1.00	12.26	11.45	3.99
	533100	Dehong	20220216	4.85	0.23	2.87	3.64	1.17	4.43	24.04	8.69
	450600	Fangchenggang	20220223	3.23	1.30	4.24	2.08	0.65	9.26	20.36	6.82
	530900	Lincang	20220223	1.91	1.60	2.26	3.08	1.13	4.24	7.39	5.84
	441900	Donggaun	20220225	1.23	1.13	5.42	0.99	0.79	42.92	6.49	3.10
	310000	Shanghai	20220226	2.89	0.28	3.24	3.13	1.05	21.58	5.28	1.53
	220200	Jilin	20220301	2.57	1.48	5.04	0.87	0.27	69.61	16.36	4.95
	370200	Qingdao	20220301	3.87	1.04	4.40	2.40	0.27	20.18	18.06	5.72
	222400	Yanbian	20220302	4.17	0.66	3.03	3.25	0.81	7.99	19.69	6.34
	220100	Changchun	20220304	1.32	2.07	5.72	1.21	0.68	27.58	8.14	3.99
	320700	Lianyungang	20220305	3.02	1.57	4.99	1.61	0.51	28.98	14.93	4.59
	330100	Hangzhou	20220305	2.87	2.03	1.84	4.67	1.23	4.24	10.41	7.36
	610100	Xi'an	20220305	3.72	0.10	2.97	3.52	1.06	6.41	14.19	6.32
	210100	Shenyang	20220306	5.07	0.86	2.24	3.87	1.07	4.13	19.73	8.28
	110000	Beijing	20220307	2.18	2.28	3.41	2.15	1.00	3.56	9.48	6.22
	120000	Tianjin	20220307	2.90	1.59	5.98	0.39	0.28	12.57	11.23	4.24
	330800	Quzhou	20220307	1.95	1.50	5.11	1.36	0.79	39.41	7.90	2.40
	371000	Weihai	20220307	2.26	0.77	4.82	1.55	0.58	20.84	5.96	2.02

	620100	Lanzhou	20220307	2.90	1.01	4.46	1.96	0.71	4.80	7.47	5.21
	130900	Cangzhou	20220308	3.36	0.54	4.23	2.18	0.74	17.66	15.04	4.76
	230100	Harbin	20220308	2.86	2.14	3.78	0.91	0.95	6.59	16.43	5.70
	370300	Zibo	20220308	3.22	1.08	4.67	2.45	0.81	10.12	6.63	2.97
	131000	Langfang	20220309	3.40	1.38	5.09	1.02	0.91	9.05	12.54	4.37
	320500	Suzhou	20220309	2.90	2.24	2.54	4.04	1.08	3.81	16.88	7.00
	220300	Siping	20220310	3.13	2.10	3.46	1.36	0.98	3.97	15.47	6.37
	320100	Nanjing	20220310	4.25	0.66	3.34	3.06	0.43	9.54	17.96	6.37
	371400	Dezhou	20220310	2.01	1.88	5.66	0.89	0.38	39.84	9.69	2.99
	330400	Jiaxing	20220311	2.51	2.55	2.55	3.17	1.05	5.97	9.29	5.81
	371600	Binzhou	20220311	1.92	3.08	4.33	1.22	0.77	27.63	10.89	6.27
	451400	Chongzuo	20220311	2.65	2.47	5.95	0.00	0.34	18.31	14.15	5.48
	500000	Chongqing	20220312	2.59	1.92	2.34	2.81	1.11	3.32	12.73	7.12
	210800	Yingkou	20220313	2.25	1.15	2.15	2.85	1.06	2.47	10.14	7.06
	320400	Changzhou	20220313	5.11	0.75	2.11	1.53	1.02	7.57	24.11	8.39
	350500	Quanzhou	20220313	4.78	1.17	2.67	3.36	0.97	2.48	18.54	7.90
	360100	Nanchang	20220313	1.93	2.15	2.37	3.46	1.13	3.66	8.67	6.58
	450700	Qinzhou	20220313	4.14	0.55	4.85	0.54	0.71	7.59	18.03	6.03
	210200	Dalian	20220314	1.88	1.89	2.88	2.66	1.04	4.70	6.89	5.36
	340700	Tongling	20220314	2.61	1.59	3.88	1.47	0.69	22.23	16.55	5.10
	371300	Linyi	20220315	3.15	2.27	3.40	2.56	0.78	5.87	14.68	6.14
	350300	Putian	20220317	3.75	1.13	4.92	0.86	0.67	8.63	17.93	5.65
	130200	Tangshan	20220319	2.81	0.47	5.34	0.98	0.76	26.36	9.88	3.24
	320600	Nantong	20220321	2.19	2.37	3.15	3.13	0.97	4.34	9.48	5.95
	411600	Zhoukou	20220322	3.00	1.71	3.47	1.09	0.94	6.58	17.03	5.83
	340200	Wuhu	20220324	1.98	1.97	5.63	0.81	0.07	6.10	8.01	4.41

	320300	Xuzhou	20220326	2.46	0.25	5.44	0.68	0.61	21.95	3.30	1.03
	330700	Jinhua	20220326	2.73	1.32	2.09	3.36	1.09	1.64	12.90	7.14
	230800	Jiamusi	20220327	3.50	1.48	4.75	1.34	0.81	6.00	16.67	5.58
	321300	Suqian	20220327	1.88	2.53	5.82	0.37	0.56	31.39	8.18	4.57
	340400	Huainan	20220327	2.19	1.13	4.24	1.31	0.77	16.28	7.97	2.96
	341200	Fuyang	20220327	2.84	1.89	3.26	1.99	0.95	2.40	9.69	6.20
	420700	Ezhou	20220327	2.99	1.90	5.15	0.21	0.14	8.46	11.38	6.00
	130600	Baoding	20220328	4.62	0.62	2.20	4.31	1.14	6.01	19.99	7.29
	330200	Ningbo	20220328	2.90	2.69	2.17	1.95	1.15	4.64	13.88	6.94
	510100	Chengdu	20220328	3.55	0.45	3.81	2.72	0.89	5.63	13.11	5.39
	220800	Baicheng	20220329	2.89	2.43	4.41	1.95	0.65	6.45	14.63	5.20
	320200	Wuxi	20220329	2.76	1.78	2.42	3.41	1.11	4.34	9.40	6.47
	370100	Jinan	20220329	2.40	1.93	5.01	1.51	0.49	5.76	13.15	4.77
	350900	Ningde	20220330	3.54	1.57	4.41	2.73	0.63	8.10	14.75	4.93
	440100	Guangzhou	20220330	2.19	1.43	6.11	0.92	0.05	12.27	6.33	4.73
	460200	Sanya	20220331	2.47	2.13	2.03	2.66	1.22	3.39	9.54	6.94
	130400	Handan	20220401	4.69	1.00	2.42	3.60	1.04	3.65	24.11	8.03
	420100	Wuhan	20220401	2.87	1.00	5.21	2.21	0.77	28.59	11.30	3.29
	341500	Lu'an	20220402	2.88	1.72	3.83	1.83	0.92	5.25	10.16	5.78
	610100	Xi'an	20220402	3.58	0.43	2.02	4.09	1.17	3.84	21.73	7.50
	140100	Langfang	20220403	3.58	0.58	4.33	2.73	0.89	11.27	10.34	3.34
	630100	Xining	20220403	3.21	1.33	2.29	3.99	1.11	4.63	12.64	6.79
	450600	Fangchenggang	20220405	3.09	0.94	3.19	2.94	1.00	10.77	5.12	2.87
	410100	Zhengzhou	20220408	2.90	1.21	2.19	3.72	1.03	9.19	19.41	6.83
	410500	Anyang	20220408	2.59	1.29	5.90	0.54	0.40	15.49	12.54	4.51
	230100	Harbin	20220413	2.98	0.56	5.10	1.76	0.17	9.15	7.65	6.05

	222400	Yanbian	20220415	2.25	0.52	3.23	2.24	1.00	16.88	5.51	1.91
	320300	Xuzhou	20220416	3.22	0.65	3.84	2.53	0.92	5.71	8.50	5.57
	130200	Tangshan	20220418	3.23	0.29	2.20	3.65	1.22	6.59	23.68	7.94
	110000	Beijing	20220420	2.15	1.83	5.23	1.76	0.03	7.39	14.55	5.66
	361100	Shangrao	20220420	2.96	0.84	4.17	1.74	0.77	9.01	7.68	4.45
	370600	Yantai	20220422	4.57	0.67	3.22	3.45	0.14	5.16	23.58	8.12
	210600	Dandong	20220424	3.08	0.10	3.45	2.73	0.75	8.26	19.70	6.61
	650100	Wulumuqi	20220427	2.01	2.07	3.03	2.79	1.04	3.76	10.61	6.26
	440100	Guangzhou	20220428	3.62	0.28	1.65	4.71	1.06	12.80	22.40	7.36
	411600	Zhoukou	20220501	3.06	0.33	2.14	2.42	1.11	6.89	19.32	6.28
	411000	Xuchang	20220503	4.34	0.60	4.45	2.52	0.67	8.59	21.65	7.35
	440800	Zhanjiang	20220506	3.11	0.45	5.61	0.69	0.16	30.31	17.77	5.80
	360900	Yichun	20220507	3.21	1.00	1.93	4.20	1.15	5.13	20.54	7.39
	511600	Guangan	20220509	3.79	1.51	2.40	3.49	1.08	5.89	21.53	7.40
	120000	Tianjin	20220512	3.28	1.44	1.98	4.57	1.20	4.38	19.54	7.51
	220600	Baishan	20220513	4.18	0.53	2.96	3.21	1.09	7.71	19.26	7.75
	210600	Dandong	20220524	2.67	1.45	2.30	4.08	1.15	3.91	7.57	6.23

D. Scenario analysis of zeroing strategies

In this study, we assessed the independent and combined effects of zeroing strategies in relation to their implementation phase and intensity. To assess the independent effect of each NPIs, our focus is on analysing the impact pattern of implementation time-point and intensity on various population-sized cities, with consideration of changes in virus transmissibility. Considering potential delays in strategy development and disease identified, we set 5, 10, 15, 20, 25 days after the index case was detected as the time

points for NPIs implementation. We focused on four categories of NPI, including social distancing measures, facial masking, PCR screening and contact tracing. Their intensities were set to increment between 0-1 with a step of 0.1. The value of contact tracing represented how many exposure individuals were controlled before they were detected. Note that each NPI was set as a separate response in the simulations to avoid the interaction of each combined option.

In view of the fact that the qualitative PCR technique was usually not available in the early stages of an emerging contagion, our study assumed some scenario to the more probable situations when assessing the NPIs combined effect. We were more concerned about which combination of social distancing measures, facial masking and contact tracing would curb the propagation in the 90 days. We conducted the simulation in which facial masking was present at an intensity of 0.25, 0.5 and 0.75, and contact tracing was combined with social distancing measures of various intensities (0, 0.25, 0.5, 0.75, 1) at 0.6, 0.7 and 0.8 intensity, respectively, with the onset of these NPIs set at days 7 and 14 of each outbreak. The criterion for evaluating the effectiveness of the combined strategies was their ability to eradicate the outbreak within the specified target time.

The NPIs implemented by cities in mainland China have varied greatly depending on the local COVID-19 situation. Some cities have implemented strict measures such as lockdowns, while others have adopted more relaxed measures. For example, a cluster of Omicron cases was reported in Beijing that was linked to a wholesale food market in January 2022. The city quickly implemented strict control measures such as mass testing, contact tracing, and targeted stay-at-home orders to contain the outbreak. However, before a large-scale outbreak occurred in February 2022, Shanghai had generally adopted

more relaxed measures compared to some other cities in China. As there were various factors that may affect the pandemic, we selected 15 cities considering their population density, healthcare capacity, the local economy, geographic location, the stringency of the measures, and even cultural diversity. These also include cities where unprecedentedly large-scale outbreaks have occurred, such as Jilin, Shanghai, Changchun, etc.

Supplementary Table 9. Basic information of 15 cities for scenario simulation. All these cities had Omicron outbreaks reported.

City category	ID	City name	Resident population (Million)
Small city	450600	Fangchenggang	104.61
	210600	Dandong	218.84
	210800	Yingkou	231.2
	340400	Huainan	304
	220200	Jilin	362.37
Medium city	140100	Taiyuan	539.1
	131000	Langfang	553.82
	361100	Shangrao	643.7
	220100	Changchun	906.69
	210100	Shenyang	911.8
Large city	370200	Qingdao	1025.67
	441900	Dongguan	1053.68
	320500	Suzhou	1284.78
	110000	Beijing	2188.6
	310000	Shanghai	2489.43

D.1 Strategies for containing future emerging infectious diseases

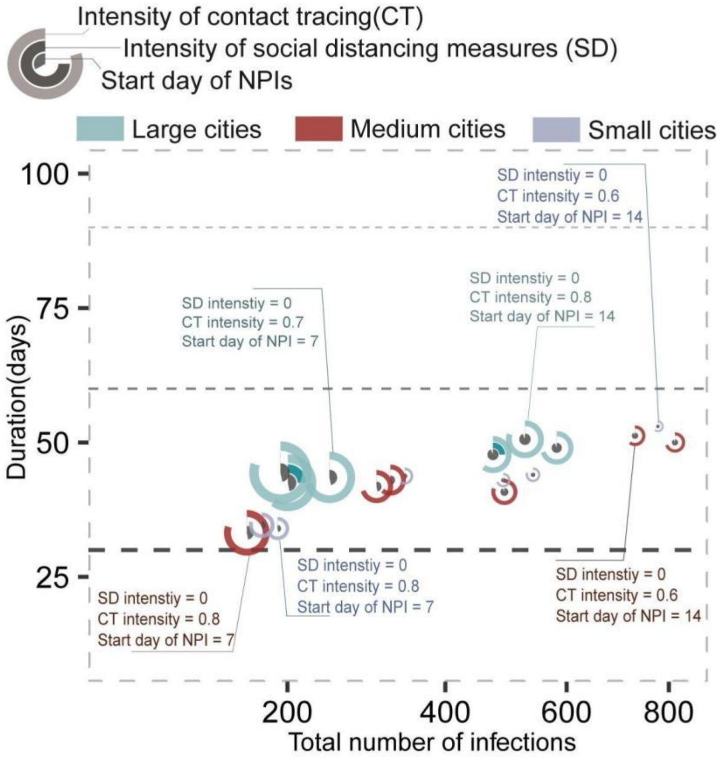

Supplementary Fig 16. Effective strategy combination options for NPIs in cities with different population sizes under R_0 = 3 and latent = 1. We assumed 50% probability that an individual wearing a mask amid contact with an infected person, considering the realizability and the generalizability to the other countries/areas**.**

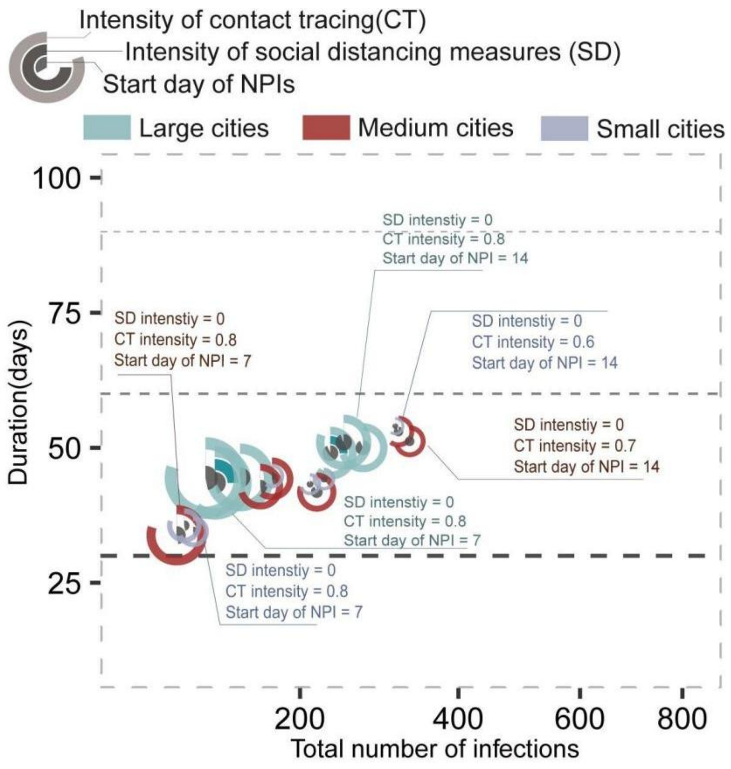

Supplementary Fig 17. Effective strategy combination options for NPIs in cities with different population sizes under R_0 = 3 and latent = 4. We assumed 50% probability that an individual wearing a mask amid contact with an infected person, considering the realizability and the generalizability to the other countries/areas

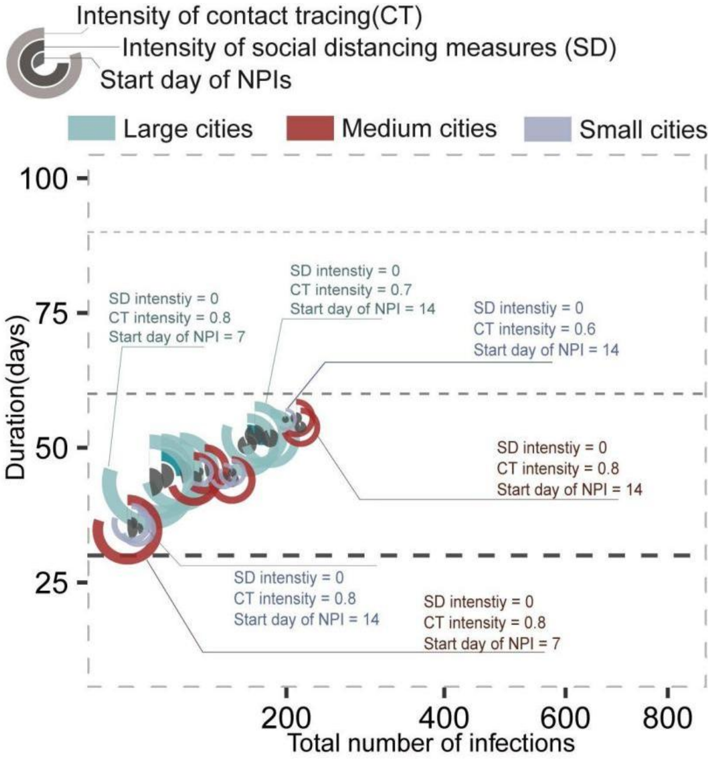

Supplementary Fig 18. Effective strategy combination options for NPIs in cities with different population sizes under R_0 = 3 and latent = 7. We assumed 50% probability that an individual wearing a mask amid contact with an infected person, considering the realizability and the generalizability to the other countries/areas

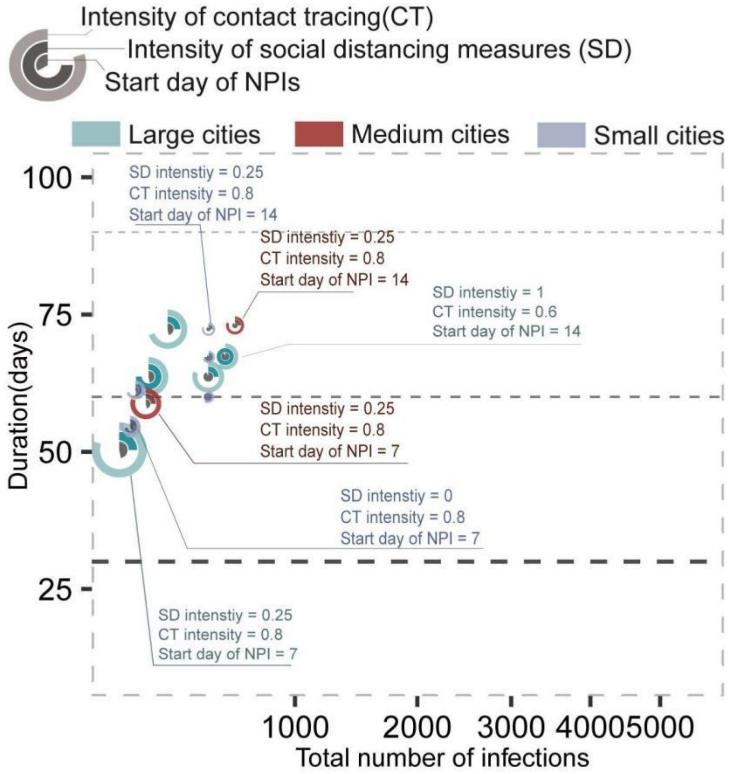

Supplementary Fig 19. Effective strategy combination options for NPIs in cities with different population sizes under R_0 = 8 and latent = 7. We assumed 50% probability that an individual wearing a mask amid contact with an infected person, considering the realizability and the generalizability to the other countries/areas

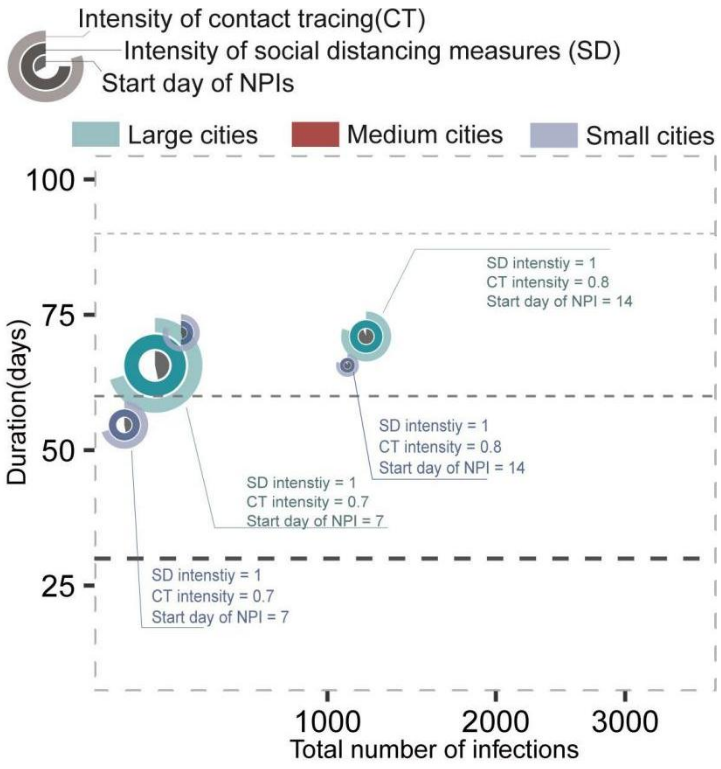

Supplementary Fig 20. Effective strategy combination options for NPIs in cities with different population sizes under R_0 = 13 and latent = 7. We assumed 50% probability that an individual wearing a mask amid contact with an infected person, considering the realizability and the generalizability to the other countries/areas.

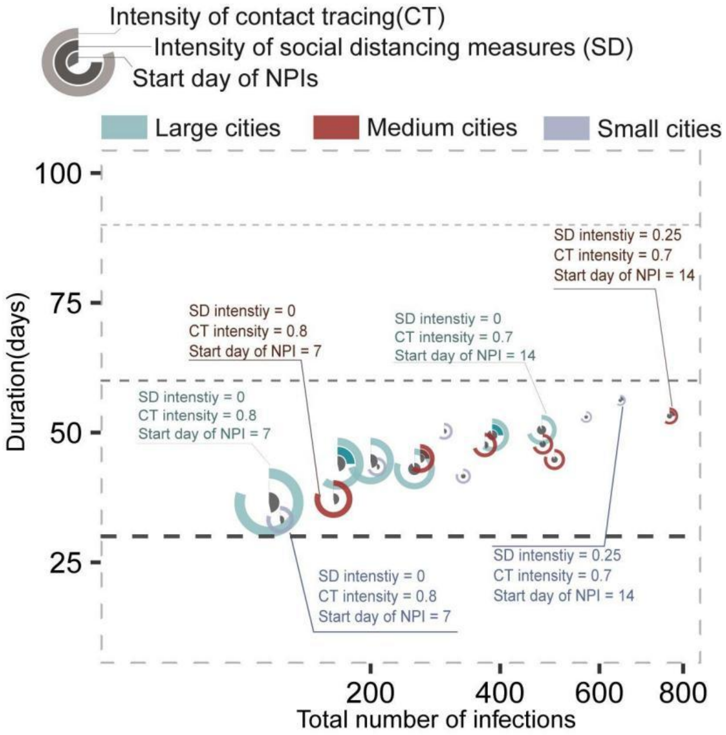

Supplementary Fig 21. Effective strategy combination options for NPIs in cities with different population sizes under R_0 = 3 and latent = 1. We assumed 25% probability that an individual wearing a mask amid contact with an infected person, considering the realizability and the generalizability to the other countries/areas.

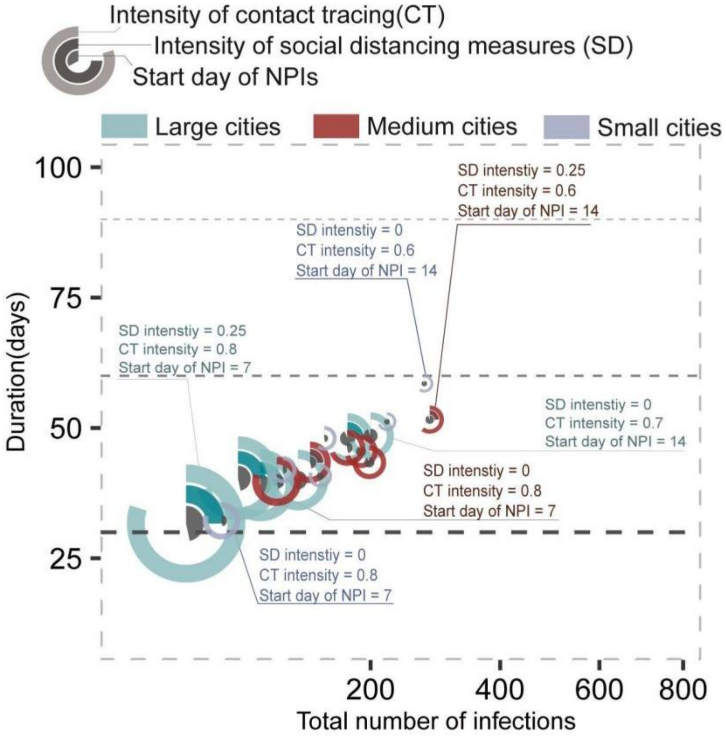

Supplementary Fig 22. Effective strategy combination options for NPIs in cities with different population sizes under R_0 = 3 and latent = 4. We assumed 25% probability that an individual wearing a mask amid contact with an infected person, considering the realizability and the generalizability to the other countries/areas.

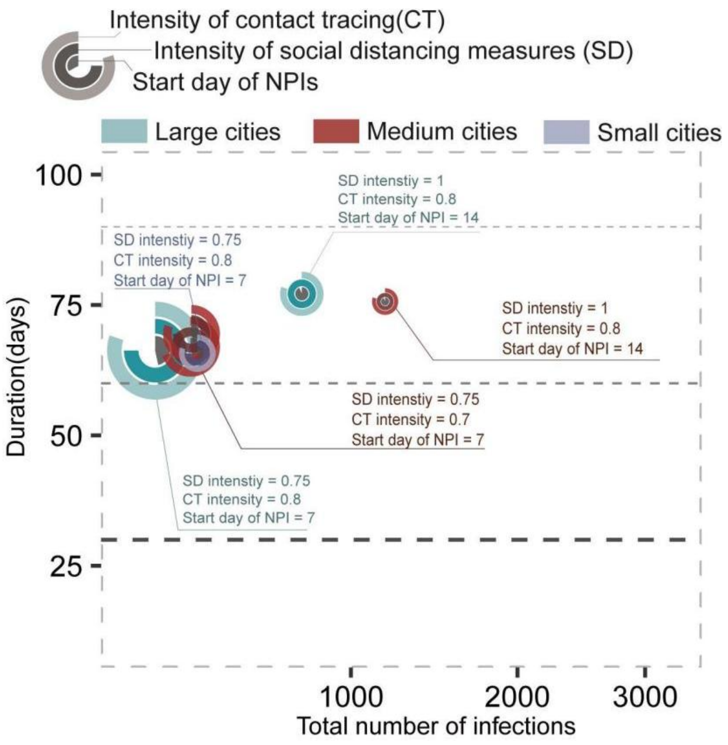

Supplementary Fig 23. Effective strategy combination options for NPIs in cities with different population sizes under R_0 = 3 and latent = 7. We assumed 25% probability that an individual wearing a mask amid contact with an infected person, considering the realizability and the generalizability to the other countries/areas.

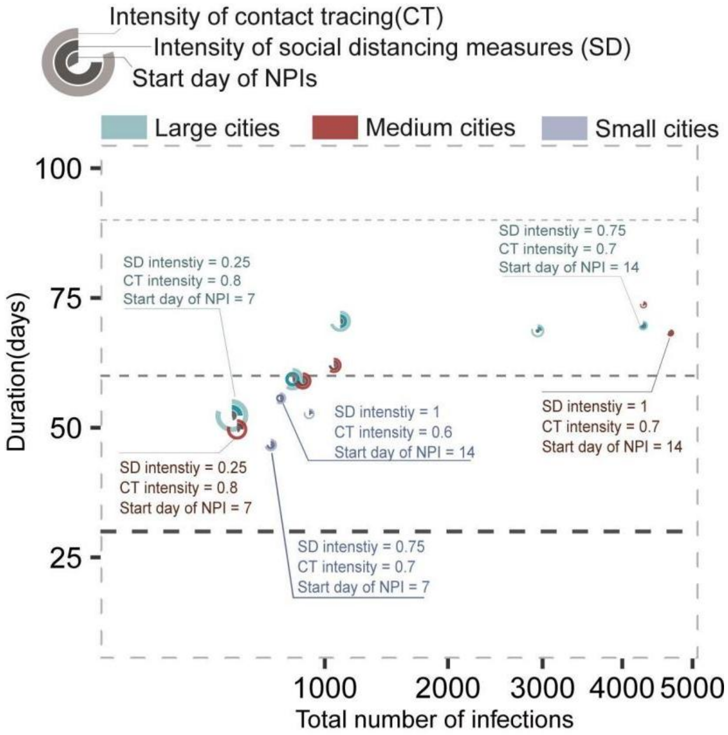

Supplementary Fig 24. Effective strategy combination options for NPIs in cities with different population sizes under R_0 = 8 and latent = 1. We assumed 25% probability that an individual wearing a mask amid contact with an infected person, considering the realizability and the generalizability to the other countries/areas.

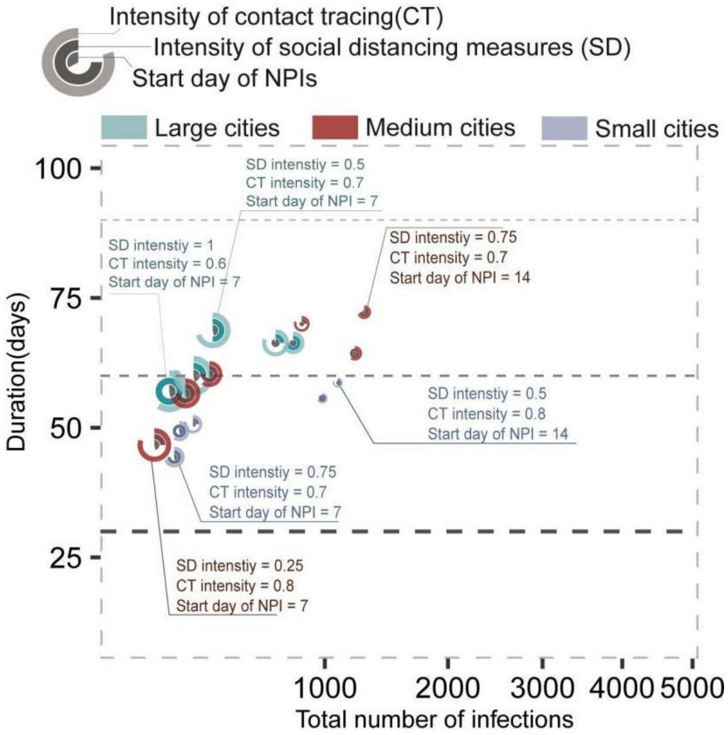

Supplementary Fig 25. Effective strategy combination options for NPIs in cities with different population sizes under R_0 = 8 and latent = 4. We assumed 25% probability that an individual wearing a mask amid contact with an infected person, considering the realizability and the generalizability to the other countries/areas.

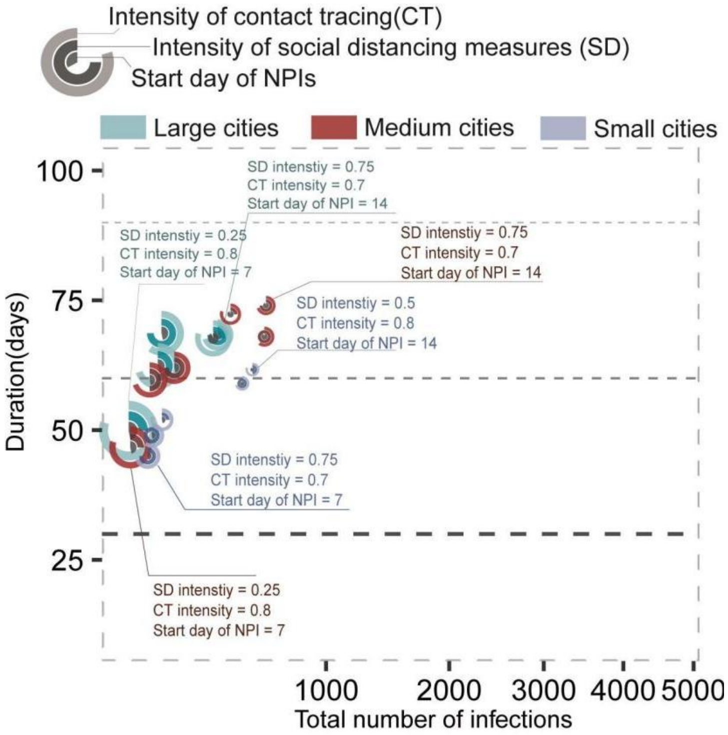

Supplementary Fig 26. Effective strategy combination options for NPIs in cities with different population sizes under R_0 = 8 and latent = 7. We assumed 25% probability that an individual wearing a mask amid contact with an infected person, considering the realizability and the generalizability to the other countries/areas.

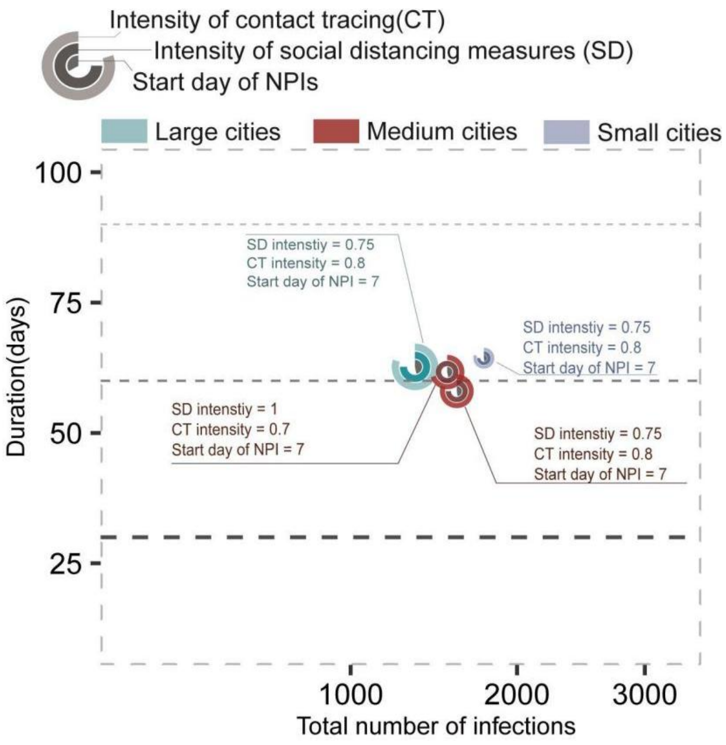

Supplementary Fig 27. Effective strategy combination options for NPIs in cities with different population sizes under R_0 = 13 and latent = 1. We assumed 25% probability that an individual wearing a mask amid contact with an infected person, considering the realizability and the generalizability to the other countries/areas.

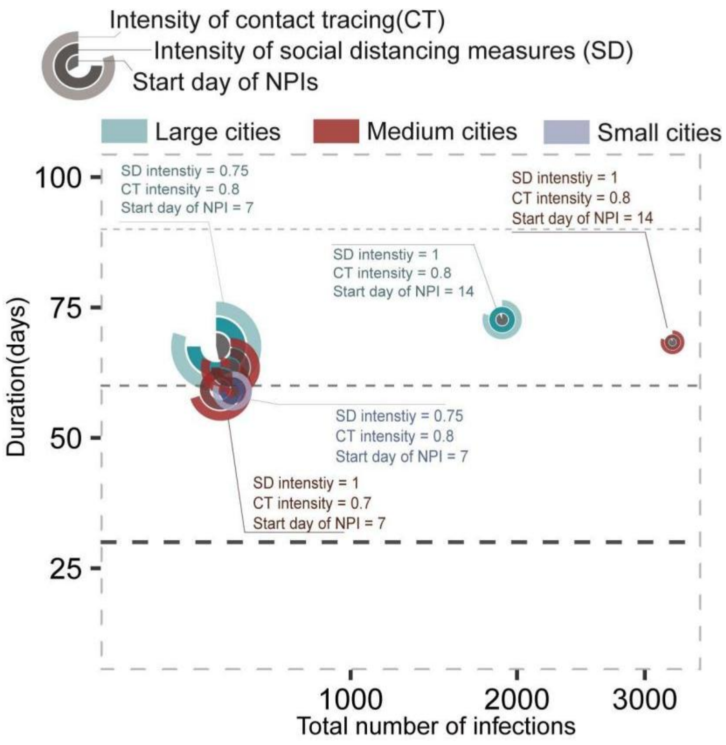

Supplementary Fig 28. Effective strategy combination options for NPIs in cities with different population sizes under R_0 = 13 and latent = 4. We assumed 25% probability that an individual wearing a mask amid contact with an infected person, considering the realizability and the generalizability to the other countries/areas.

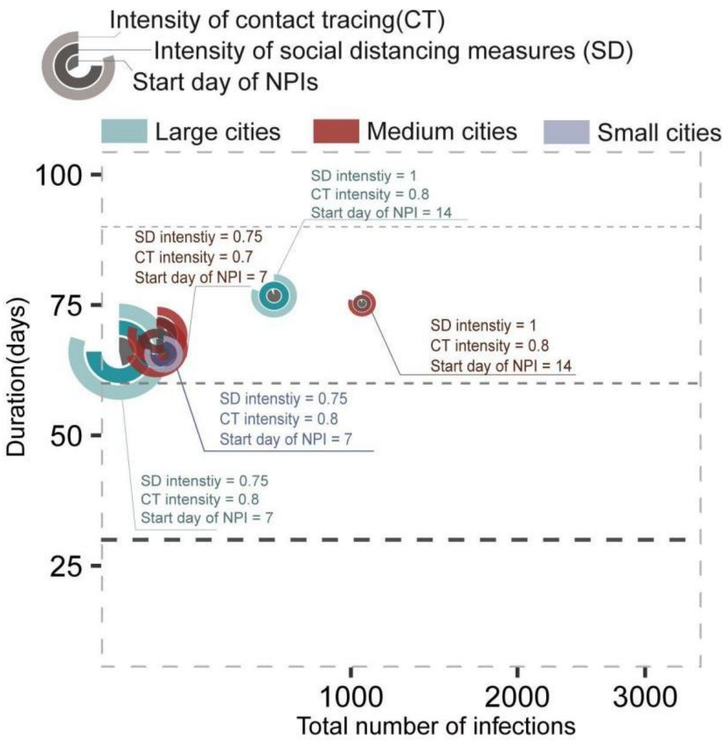

Supplementary Fig 29. Effective strategy combination options for NPIs in cities with different population sizes under R_0 = 13 and latent = 7. We assumed 25% probability that an individual wearing a mask amid contact with an infected person, considering the realizability and the generalizability to the other countries/areas.

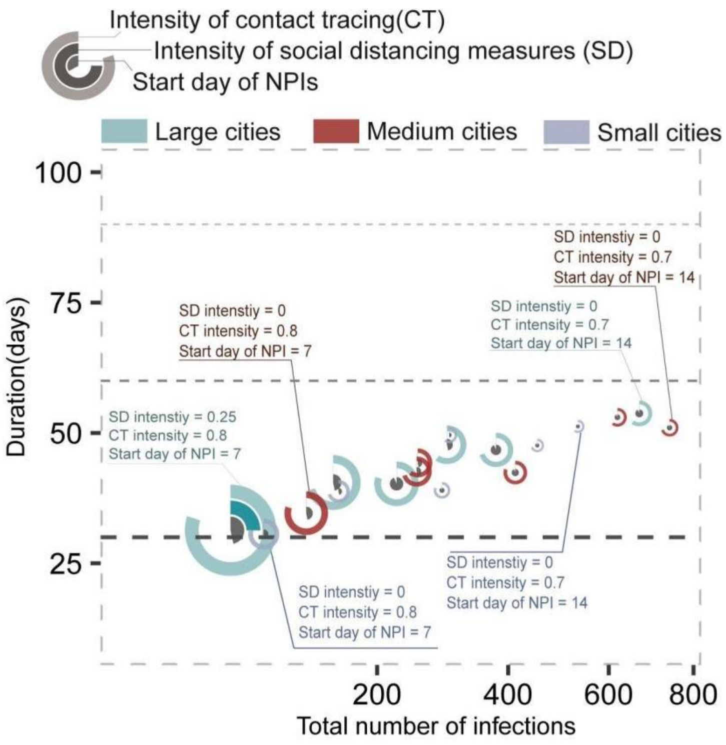

Supplementary Fig 30. Effective strategy combination options for NPIs in cities with different population sizes under R_0 = 3 and latent = 1. We assumed 75% probability that an individual wearing a mask amid contact with an infected person, considering the realizability and the generalizability to the other countries/areas.

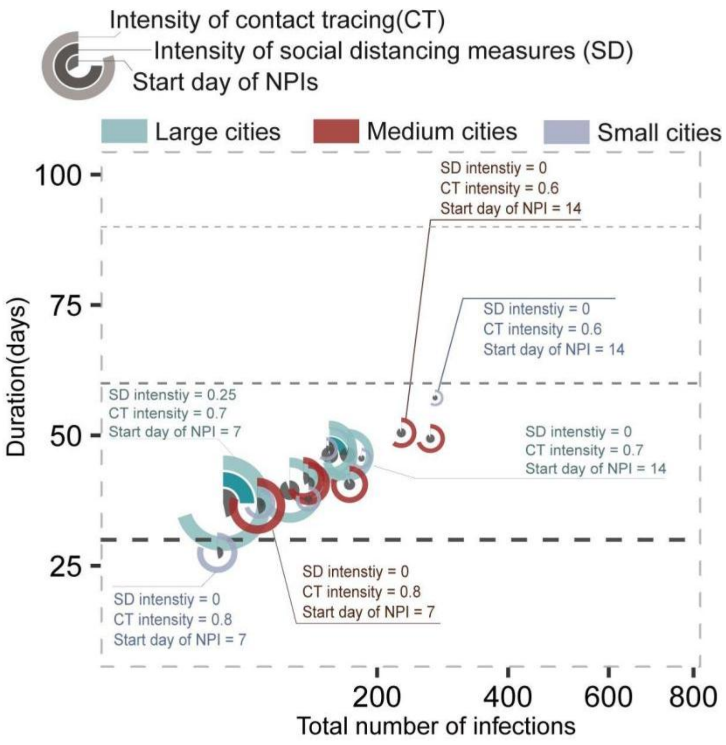

Supplementary Fig 31. Effective strategy combination options for NPIs in cities with different population sizes under R_0 = 3 and latent = 4. We assumed 75% probability that an individual wearing a mask amid contact with an infected person, considering the realizability and the generalizability to the other countries/areas.

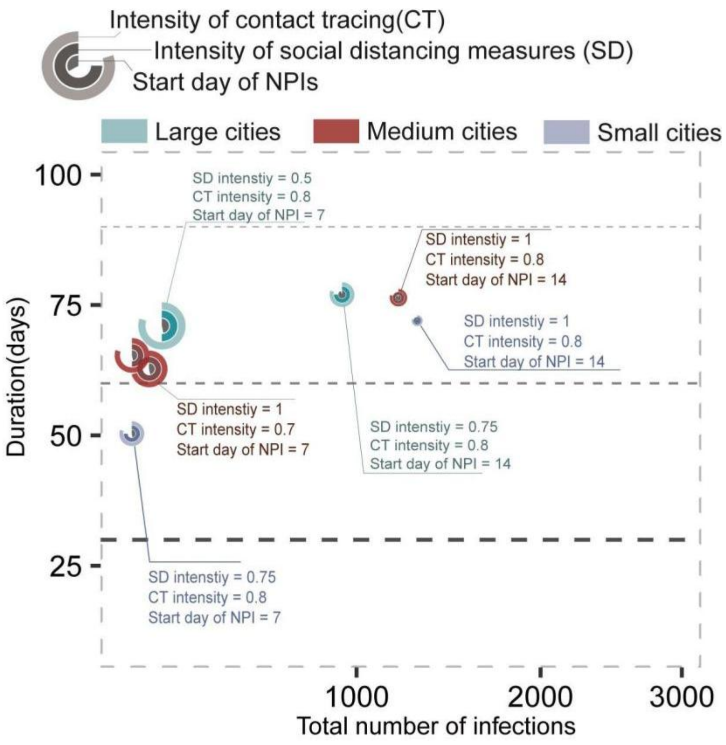

Supplementary Fig 32. Effective strategy combination options for NPIs in cities with different population sizes under R_0 = 3 and latent = 7. We assumed 75% probability that an individual wearing a mask amid contact with an infected person, considering the realizability and the generalizability to the other countries/areas.

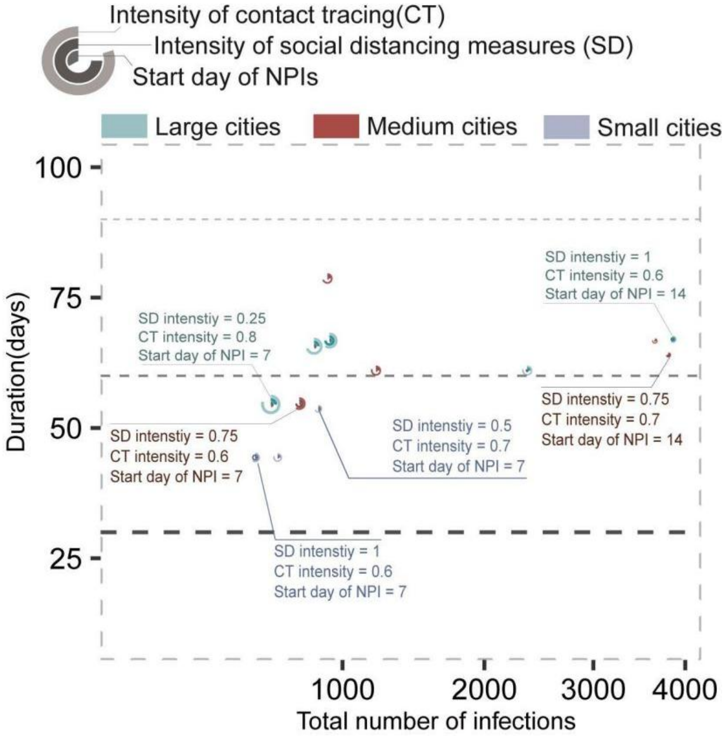

Supplementary Fig 33. Effective strategy combination options for NPIs in cities with different population sizes under R_0 = 8 and latent = 1. We assumed 75% probability that an individual wearing a mask amid contact with an infected person, considering the realizability and the generalizability to the other countries/areas.

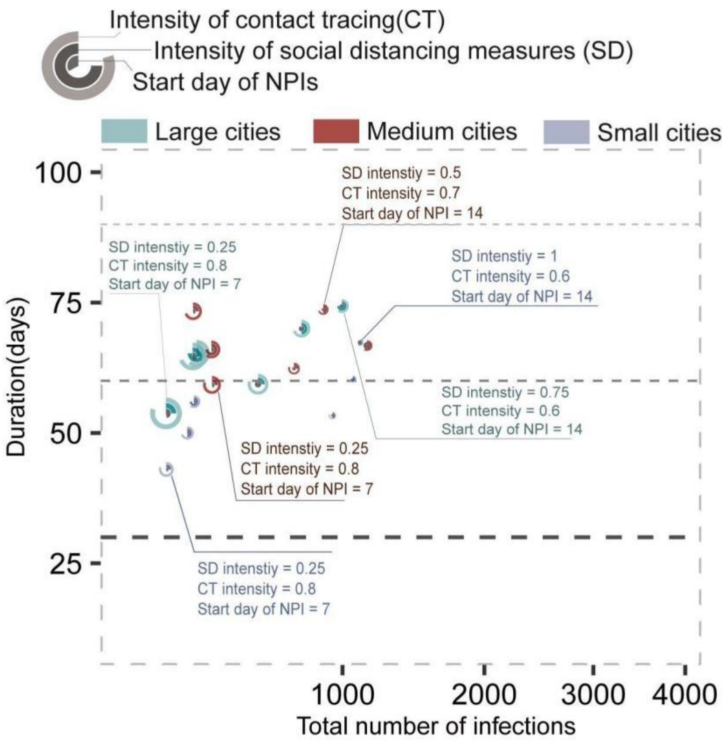

Supplementary Fig 34. Effective strategy combination options for NPIs in cities with different population sizes under R_0 = 8 and latent = 4. We assumed 75% probability that an individual wearing a mask amid contact with an infected person, considering the realizability and the generalizability to the other countries/areas.

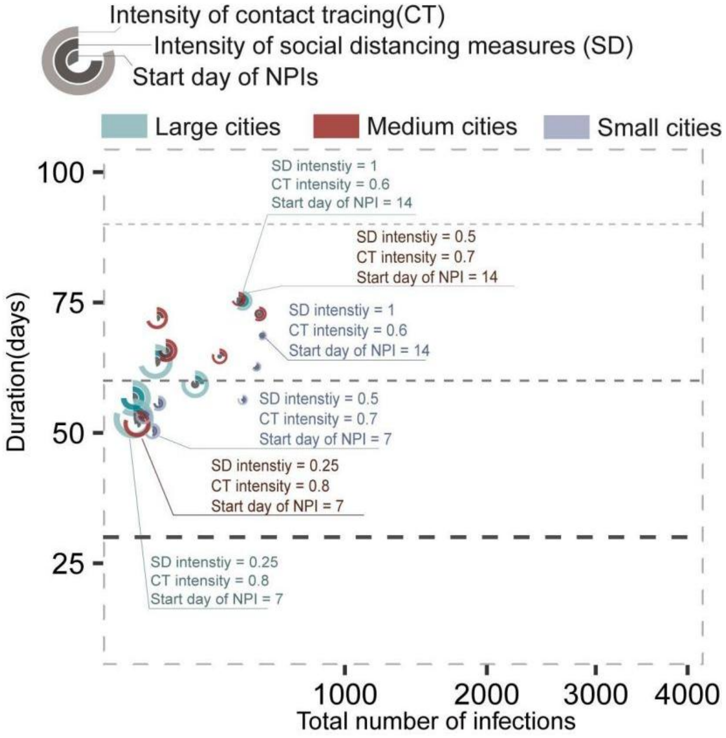

Supplementary Fig 35. Effective strategy combination options for NPIs in cities with different population sizes under R_0 = 8 and latent = 7. We assumed 75% probability that an individual wearing a mask amid contact with an infected person, considering the realizability and the generalizability to the other countries/areas.

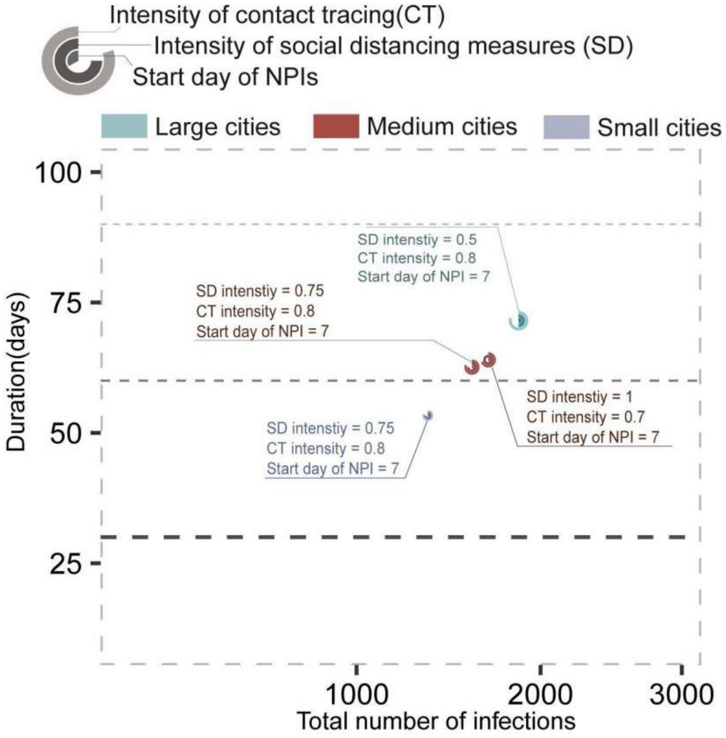

Supplementary Fig 36. Effective strategy combination options for NPIs in cities with different population sizes under R_0 = 13 and latent = 1. We assumed 75% probability that an individual wearing a mask amid contact with an infected person, considering the realizability and the generalizability to the other countries/areas.

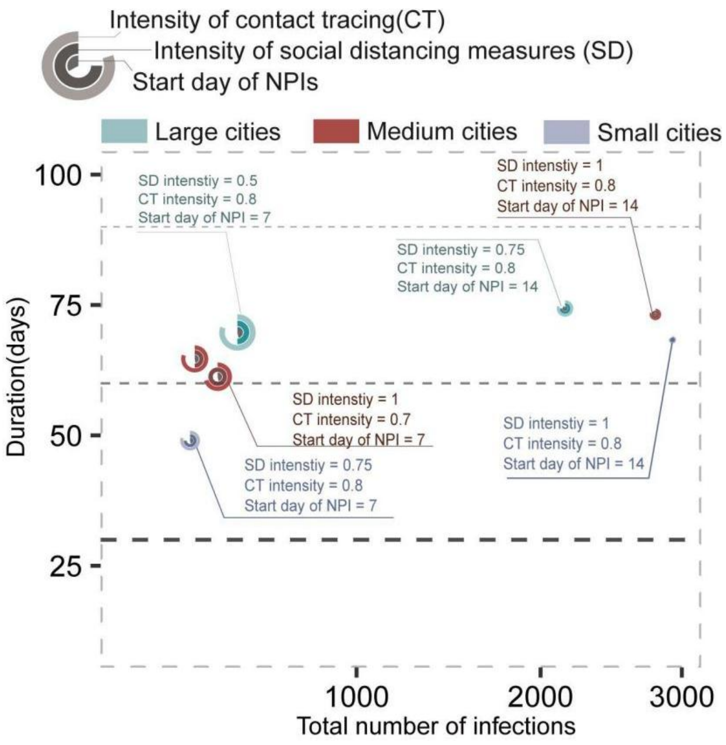

Supplementary Fig 37. Effective strategy combination options for NPIs in cities with different population sizes under R_0 = 13 and latent = 4. We assumed 75% probability that an individual wearing a mask amid contact with an infected person, considering the realizability and the generalizability to the other countries/areas.

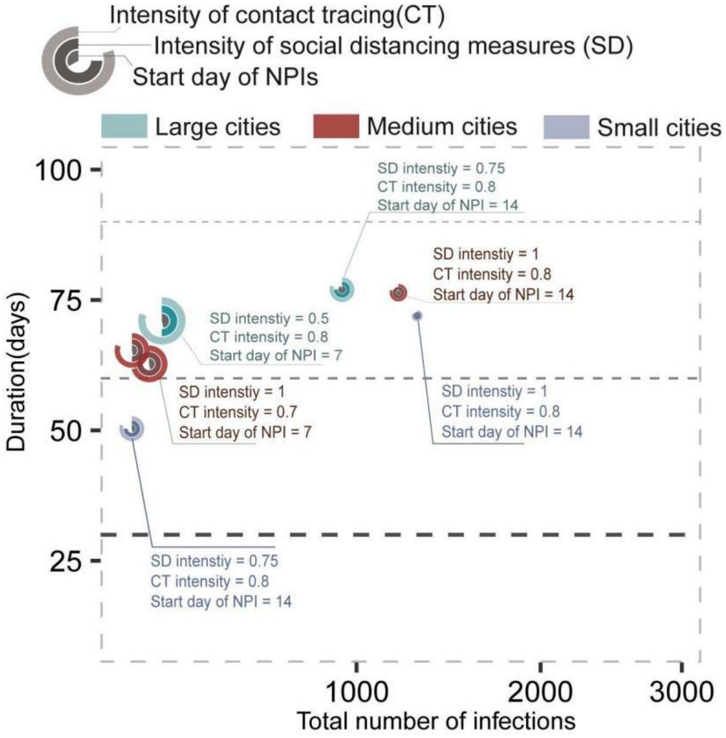

Supplementary Fig 38. Effective strategy combination options for NPIs in cities with different population sizes under R_0 = 13 and latent = 7. We assumed 75% probability that an individual wearing a mask amid contact with an infected person, considering the realizability and the generalizability to the other countries/areas.

E. Sensitivity analysis

In order to evaluate the robustness of our Bayesian inference model, we designed several sensitivity analyses using different settings. These sensitivity analyses were designed to explore the impact of different model assumptions on the estimated effectiveness of NPIs in controlling the spread of Covid-19 in China. Specifically, we designed four scenarios (BS1-BS4 and DBS) that varied the prior distribution of the reproduction number (R_t) and the prior variance of city-specific characteristics effect.

- BS1. The prior distribution of R_t was set as Weibull distribution,

- BS2. The prior distribution of R_t was set as normal distribution,
- BS3. The prior variance of city-specific characteristics effect was set as 0.4,
- BS4. The prior variance of city-specific characteristics effect was set as 0.6.
- DBS. The prior distribution of R_t was set as Gamma distribution, while the prior variance of city-specific characteristics effect was set as 0.5.

The results of our sensitivity analyses showed that the outputs of the Bayesian inference model were highly sensitive to the setting of the prior distribution of R_t, rather than the prior variance of city-specific characteristics effect (see supplementary Figure 39). In particular, we found that the choice of prior distribution for R_t had a much greater impact on the estimated effect. Given these findings, we decided to set the prior distribution of R_t as a gamma distribution in our main analysis, as this prior distribution is more commonly used in related research[29–32].

By conducting sensitivity analyses and carefully selecting our model settings, we are confident in the validity and reliability of our Bayesian inference model for estimating the effectiveness of NPIs. For ISEIRV model, we designed three scenarios below, and these results showed that the model was robust and consistent.

- IS1. The efficiency of facial masking for preventing indoor transmission was set as 0.1,
- IS2. The efficiency of facial masking for preventing indoor transmission was set as 0.3,
- IS3. The efficiency of facial masking for preventing indoor transmission was set as 0.5.

- DIS. The efficiency of facial masking for preventing indoor transmission was set as 0.25.

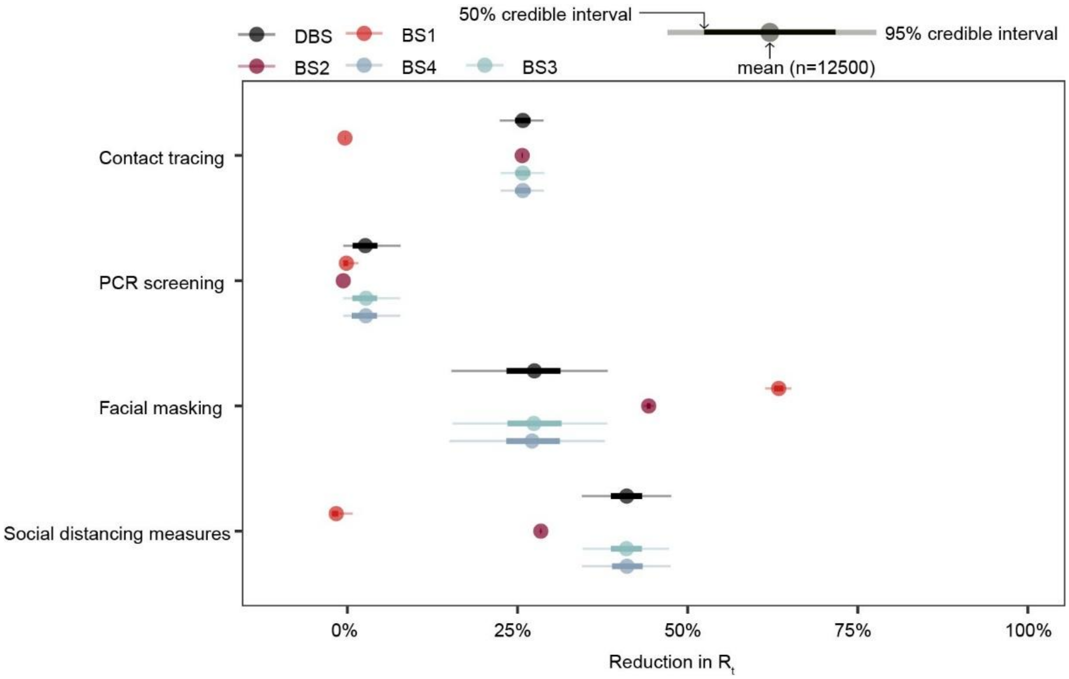

Supplementary Fig 39. Overall effects of social distance management, facial masking, PCR screening and contact tracing on reducing COVID-19 transmission under different model settings.

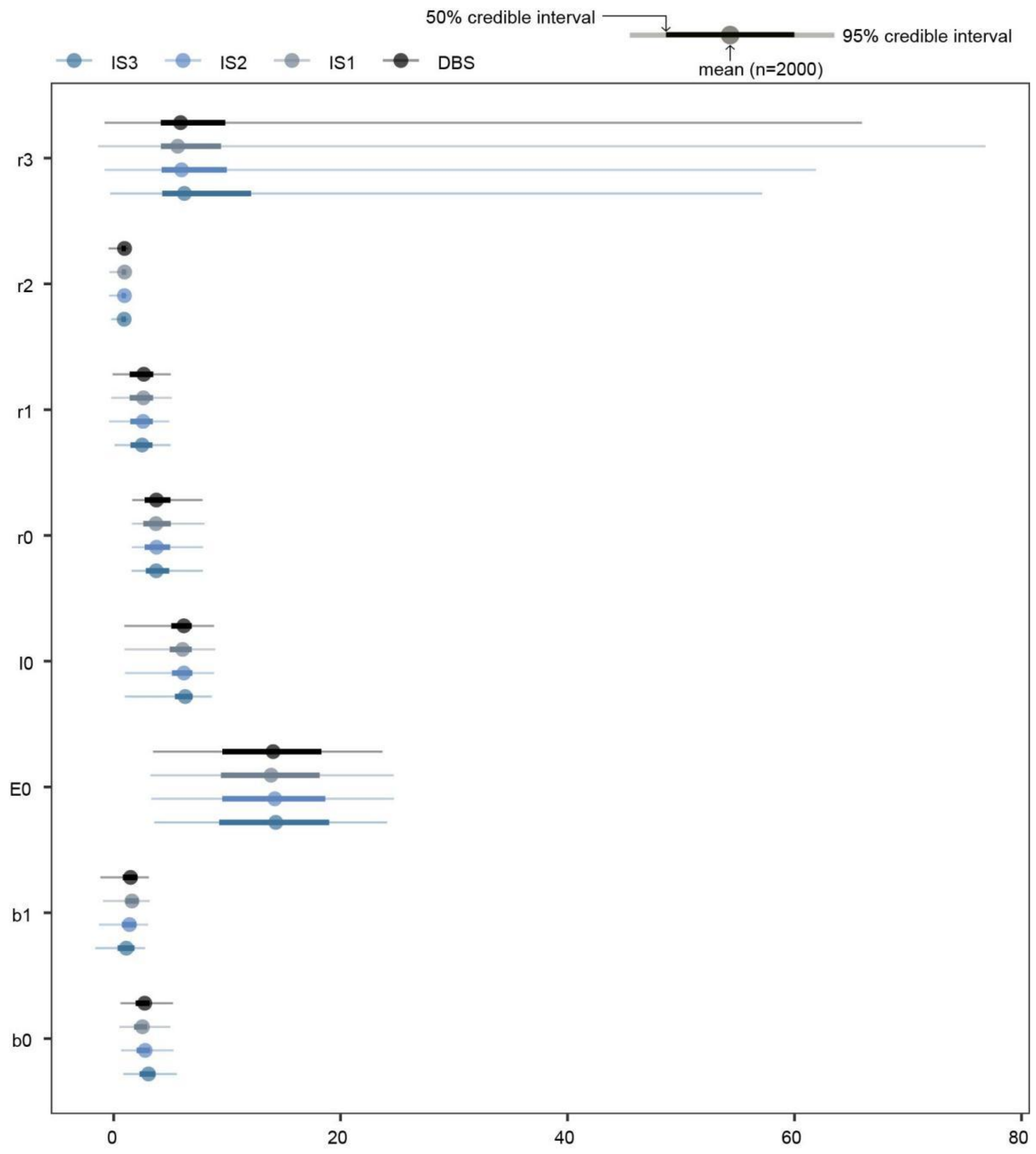

Supplementary Fig 40. Estimated values of parameters for ISEIRV model. b_0 indicates the baseline of the transmission rate. r_0 indicates the baseline of recovery period. b_1 is the effect of contact related NPIs on reducing individual-level contact frequency. r_1 is the effect of the infectious detection related NPIs on improving detection rate. r_2 and r_3 are coefficients to portray the policy lag. $E(0)$ indicates the initial exposed population. $I(0)$ indicates the initial infectious population.

Reference

1. Chinazzi, M. *et al.* The effect of travel restrictions on the spread of the 2019 novel coronavirus (COVID-19) outbreak. *Science (1979)* **368**, 395–400 (2020).
2. Zhu, P. & Guo, Y. The role of high-speed rail and air travel in the spread of COVID-19 in China. *Travel Med Infect Dis* **42**, 102097 (2021).
3. Brauner, J. M. *et al.* Inferring the effectiveness of government interventions against COVID-19. *Science (1979)* **371**, (2021).
4. Banholzer, N. *et al.* Estimating the effects of non-pharmaceutical interventions on the number of new infections with COVID-19 during the first epidemic wave. *PLoS One* **16**, e0252827 (2021).
5. Flaxman, S. *et al.* Estimating the effects of non-pharmaceutical interventions on COVID-19 in Europe. *Nature* **584**, 257–261 (2020).
6. Bo, Y. *et al.* Effectiveness of non-pharmaceutical interventions on COVID-19 transmission in 190 countries from 23 January to 13 April 2020. *International Journal of Infectious Diseases* **102**, 247–253 (2021).
7. Rees, E. *et al.* Effectiveness of non-pharmaceutical interventions to reduce SARS-CoV-2 transmission in Canada and their association with COVID-19 hospitalization rates. *Canada Communicable Disease Report* **48**, 438–448 (2022).
8. Zheng, W., Yan, X., Zhao, Z., Yang, J. & Yu, H. COVID-19 vaccination program in the mainland of China: a subnational descriptive analysis on target population size and current progress. *Infect Dis Poverty* **10**, 124 (2021).
9. Chin, E. T. *et al.* Protection against Omicron from Vaccination and Previous Infection in a Prison System. *New England Journal of Medicine* **387**, 1770–1782 (2022).
10. Andrews, N. *et al.* Covid-19 Vaccine Effectiveness against the Omicron (B.1.1.529) Variant. *New England Journal of Medicine* **386**, 1532–1546 (2022).
11. World Health Organization. *Interim recommendations for use of the inactivated COVID-19 vaccine, CoronaVac, developed by Sinovac: interim guidance, first issued 24 May 2021, updated 21 October 2021, updated 15 March 2022*. https://apps.who.int/iris/handle/10665/352472 (2022).
12. World Health Organization. *Interim recommendations for use of the inactivated COVID-19 vaccine BIBP developed by China National Biotec Group (CNBG), Sinopharm: interim guidance, first issued 7 May 2021, updated 28 October 2021, updated 15 March 2022*. https://apps.who.int/iris/handle/10665/352470 (2022).
13. Lai, S. *et al.* Effect of non-pharmaceutical interventions to contain COVID-19 in China. *Nature 2020 585:7825* **585**, 410–413 (2020).
14. Li, Z. *et al.* Active case finding with case management: the key to tackling the COVID-19 pandemic. *Lancet* **396**, 63–70 (2020).
15. Liu, J. *et al.* The Dynamic COVID-Zero Strategy in China. *China CDC Weekly, 2022, Vol. 4, Issue 4, Pages: 74-75* **4**, 74–75 (2022).
16. Anne Cori *et al.* Package 'EpiEstim'. Preprint at https://cran.r-project.org/web/packages/EpiEstim/index.html (2021).
17. Alene, M. *et al.* Serial interval and incubation period of COVID-19: a systematic review and meta-analysis. *BMC Infect Dis* **21**, 257 (2021).

18. Geismar, C. *et al.* Household serial interval of COVID-19 and the effect of Variant B.1.1.7: analyses from prospective community cohort study (Virus Watch). *Wellcome Open Res* **6**, 224 (2021).
19. Zhang, M. *et al.* Transmission Dynamics of an Outbreak of the COVID-19 Delta Variant B.1.617.2 — Guangdong Province, China, May–June 2021. *China CDC Wkly* **3**, 584–586 (2021).
20. Song, J. S. *et al.* Serial Intervals and Household Transmission of SARS-CoV-2 Omicron Variant, South Korea, 2021. *Emerg Infect Dis* **28**, 756–759 (2022).
21. Paul, S. & Lorin, E. Distribution of incubation periods of COVID-19 in the Canadian context. *Sci Rep* **11**, 12569 (2021).
22. Tanaka, H. *et al.* Shorter Incubation Period among COVID-19 Cases with the BA.1 Omicron Variant. *Int J Environ Res Public Health* **19**, 6330 (2022).
23. Ogata, T., Tanaka, H., Irie, F., Hirayama, A. & Takahashi, Y. Shorter Incubation Period among Unvaccinated Delta Variant Coronavirus Disease 2019 Patients in Japan. *Int J Environ Res Public Health* **19**, 1127 (2022).
24. Alimohamadi, Y., Taghdir, M. & Sepandi, M. Estimate of the Basic Reproduction Number for COVID-19: A Systematic Review and Meta-analysis. *Journal of Preventive Medicine and Public Health* **53**, 151–157 (2020).
25. Campbell, F. *et al.* Increased transmissibility and global spread of SARS-CoV-2 variants of concern as at June 2021. *Eurosurveillance* **26**, (2021).
26. Kang, M. *et al.* Transmission dynamics and epidemiological characteristics of SARS-CoV-2 Delta variant infections in Guangdong, China, May to June 2021. *Eurosurveillance* **27**, (2022).
27. Liu, Y. & Rocklöv, J. The effective reproductive number of the Omicron variant of SARS-CoV-2 is several times relative to Delta. *J Travel Med* **29**, (2022).
28. Wang, J.-F., Zhang, T.-L. & Fu, B.-J. A measure of spatial stratified heterogeneity. *Ecol Indic* **67**, 250–256 (2016).
29. Routledge, I. *et al.* Estimating spatiotemporally varying malaria reproduction numbers in a near elimination setting. *Nat Commun* **9**, 2476 (2018).
30. Huisman, J. S. *et al.* Estimation and worldwide monitoring of the effective reproductive number of SARS-CoV-2. *Elife* **11**, (2022).
31. Ge, Y. *et al.* Untangling the changing impact of non-pharmaceutical interventions and vaccination on European COVID-19 trajectories. *Nat Commun* **13**, 3106 (2022).
32. Cori, A., Ferguson, N. M., Fraser, C. & Cauchemez, S. A New Framework and Software to Estimate Time-Varying Reproduction Numbers During Epidemics. *Am J Epidemiol* **178**, 1505–1512 (2013).
