# Supplementary material for: Effects of public-health measures for zeroing out different SARS-CoV-2 variants
Source: Nat Commun. 2023 Aug 29;14:5270. doi: 10.1038/s41467-023-40940-4 (PMC10465600; doi:10.1038/s41467-023-40940-4)
Supplement: Supplementary file 3 — Reporting Summary [file 41467_2023_40940_MOESM3_ESM.pdf]

nature portfolio

Corresponding author(s): Yong Ge & Shengjie Lai

Last updated by author(s): Aug 3, 2023

Reporting Summary

Nature Portfolio wishes to improve the reproducibility of the work that we publish. This form provides structure for consistency and transparency in reporting. For further information on Nature Portfolio policies, see our Editorial Policies and the Editorial Policy Checklist.

Statistics

For all statistical analyses, confirm that the following items are present in the figure legend, table legend, main text, or Methods section.

n/a	Confirmed	
☐	☒	The exact sample size (*n*) for each experimental group/condition, given as a discrete number and unit of measurement
☐	☒	A statement on whether measurements were taken from distinct samples or whether the same sample was measured repeatedly
☐	☒	The statistical test(s) used AND whether they are one- or two-sided<br>*Only common tests should be described solely by name; describe more complex techniques in the Methods section.*
☐	☒	A description of all covariates tested
☐	☒	A description of any assumptions or corrections, such as tests of normality and adjustment for multiple comparisons
☐	☒	A full description of the statistical parameters including central tendency (e.g. means) or other basic estimates (e.g. regression coefficient) AND variation (e.g. standard deviation) or associated estimates of uncertainty (e.g. confidence intervals)
☐	☒	For null hypothesis testing, the test statistic (e.g. *F*, *t*, *r*) with confidence intervals, effect sizes, degrees of freedom and *P* value noted<br>*Give P values as exact values whenever suitable.*
☐	☒	For Bayesian analysis, information on the choice of priors and Markov chain Monte Carlo settings
☒	☐	For hierarchical and complex designs, identification of the appropriate level for tests and full reporting of outcomes
☐	☒	Estimates of effect sizes (e.g. Cohen's *d*, Pearson's *r*), indicating how they were calculated

Our web collection on statistics for biologists contains articles on many of the points above.

Software and code

Policy information about availability of computer code

Data collection	The code for processing climate data on GEE can be found via https://code.earthengine.google.com/81f7cd2ef122d3f7f597a63cc603196d.
Data analysis	R version 4.1.1 (R Foundation for Statistical Computing, Vienna, Austria) and Matlab version R2022a were used to perform data collation and analyses. The R packages used in this study for data analysis are 'EpiEstim' with version 2.2-4 and 'rstan' with version 2.21.5. The model built by this study has been made openly available for further use at https://github.com/wxl1379457192/Zeroing_out_emerging_contagions.

For manuscripts utilizing custom algorithms or software that are central to the research but not yet described in published literature, software must be made available to editors and reviewers. We strongly encourage code deposition in a community repository (e.g. GitHub). See the Nature Portfolio guidelines for submitting code & software for further information.

Data

Policy information about availability of data

All manuscripts must include a data availability statement. This statement should provide the following information, where applicable:

- Accession codes, unique identifiers, or web links for publicly available datasets
- A description of any restrictions on data availability
- For clinical datasets or third party data, please ensure that the statement adheres to our policy

The data and code used in this study are publicly available online at https://github.com/wxl1379457192/Zeroing_out_emerging_contagions.

Human research participants

Policy information about studies involving human research participants and Sex and Gender in Research.

Reporting on sex and gender	Not applicable
Population characteristics	Not applicable
Recruitment	Not applicable
Ethics oversight	Ethical clearance for collecting and using secondary data in this study was granted by the institutional review board of the University of Southampton (No. 61865). All data were supplied and analysed in an anonymous format, without access to personal identifying information.

Note that full information on the approval of the study protocol must also be provided in the manuscript.

Field-specific reporting

Please select the one below that is the best fit for your research. If you are not sure, read the appropriate sections before making your selection.

☐ Life sciences ☒ Behavioural & social sciences ☐ Ecological, evolutionary & environmental sciences

For a reference copy of the document with all sections, see nature.com/documents/nr-reporting-summary-flat.pdf

Behavioural & social sciences study design

All studies must disclose on these points even when the disclosure is negative.

Study description	Quantitative observational and modelling study
Research sample	Epidemiological data, vaccinated population, timing and intensity of non-pharmaceutical interventions and city-specific characteristics reported across 262 outbreaks in the mainland of China from 1 April 2020 to 31, May, 2022. The daily reports of COVID-19 cases and suspected sources, start and end dates, and strains of 261 outbreaks were collected from local government websites, reports from the local Centers for Disease Control and Prevention, as well as updates from official social media accounts of local authorities or health departments. To ensure sufficient sample size for assessing intervention effects, we excluded outbreaks with fewer than 50 cases or durations of less than 7 days. Ultimately, a total of 131 outbreaks were employed. Further, we also collected the daily data of public-health measures from the websites of local governments. The climate data was collated from the Global Land Data Assimilation System (https://developers.google.com/earth-engine/datasets/catalog/NASA_GLDAS_V021_NOAH_G025_T3H). Vaccination data were collected at province level from http://www.nhc.gov.cn/.
Sampling strategy	The number of datasets involved in this study was determined by the study object. In addition to the necessary epidemiological data, vaccination data, timing and intensity of non-pharmaceutical interventions datasets, variant structure dataset was used to define the COVID-19 transmission contexts across cities, the population and climate dataset was used to explain the different environmental conditions across cities.
Data collection	All the data used in this study are publicly available from data sources and processed in our manuscript by R and Matlab. The researchers were not blinded to experimental condition.
Timing	April 1. 2020 - May 31, 2022.
Data exclusions	To ensure sufficient sample size for assessing intervention effects, we ruled out the outbreaks that had less than 50 cases or lasted less than 7 days.
Non-participation	No participants were involved in the study.
Randomization	No applicable. The leave-one-out cross validation method was used to validate our model. We also performed a sensitivity analysis to assess the robustness of model parameter assumptions.

Reporting for specific materials, systems and methods

We require information from authors about some types of materials, experimental systems and methods used in many studies. Here, indicate whether each material, system or method listed is relevant to your study. If you are not sure if a list item applies to your research, read the appropriate section before selecting a response.

Materials & experimental systems

n/a	Involved in the study	
☒	☐	Antibodies
☒	☐	Eukaryotic cell lines
☒	☐	Palaeontology and archaeology
☒	☐	Animals and other organisms
☒	☐	Clinical data
☒	☐	Dual use research of concern

Methods

n/a	Involved in the study	
☒	☐	ChIP-seq
☒	☐	Flow cytometry
☒	☐	MRI-based neuroimaging
